# The metabolome as a diagnostic for maximal aerobic capacity during exercise in type 1 diabetes

**DOI:** 10.1007/s00125-024-06153-0

**Published:** 2024-04-25

**Authors:** Guy S. Taylor, Kieran Smith, Jadine Scragg, Timothy J. McDonald, James A. Shaw, Daniel J. West, Lee D. Roberts

**Affiliations:** 1 Human Nutrition & Exercise Research Centre, Population Health Sciences Institute, Newcastle University, Newcastle upon Tyne, UK; 2 The Oxford Centre for Diabetes, Endocrinology and Metabolism, Churchill Hospital, University of Oxford, Oxford, UK; 3 Nuffield Department of Primary Care Health Sciences, University of Oxford, Oxford, UK; 4 University of Exeter Medical School, University of Exeter, Exeter, UK; 5 Translational and Clinical Research Institute, Newcastle University, Newcastle upon Tyne, UK; 6 Leeds Institute of Cardiovascular and Metabolic Medicine, University of Leeds, Leeds, UK

**Keywords:** Aerobic exercise, Diagnostic, Metabolism, Targeted metabolomics, Type 1 diabetes

## Abstract

**Aims/hypothesis:**

Our aim was to characterise the in-depth metabolic response to aerobic exercise and the impact of residual pancreatic beta cell function in type 1 diabetes. We also aimed to use the metabolome to distinguish individuals with type 1 diabetes with reduced maximal aerobic capacity in exercise defined by $\dot{V}{\text{O}}_{\text{2peak}}$.

**Methods:**

Thirty participants with type 1 diabetes (≥3 years duration) and 30 control participants were recruited. Groups did not differ in age or sex. After quantification of peak stimulated C-peptide, participants were categorised into those with undetectable (<3 pmol/l), low (3–200 pmol/l) or high (>200 pmol/l) residual beta cell function. Maximal aerobic capacity was assessed by $\dot{V}{\text{O}}_{\text{2peak}}$ test and did not differ between control and type 1 diabetes groups. All participants completed 45 min of incline treadmill walking (60% $\dot{V}{\text{O}}_{\text{2peak}}$) with venous blood taken prior to exercise, immediately post exercise and after 60 min recovery. Serum was analysed using targeted metabolomics. Metabolomic data were analysed by multivariate statistics to define the metabolic phenotype of exercise in type 1 diabetes. Receiver operating characteristic (ROC) curves were used to identify circulating metabolomic markers of maximal aerobic capacity ($\dot{V}{\text{O}}_{\text{2peak}}$) during exercise in health and type 1 diabetes.

**Results:**

Maximal aerobic capacity ($\dot{V}{\text{O}}_{\text{2peak}}$) inversely correlated with HbA_1c_ in the type 1 diabetes group (*r*^*2*^=0.17, *p*=0.024). Higher resting serum tricarboxylic acid cycle metabolites malic acid (fold change 1.4, *p*=0.001) and lactate (fold change 1.22, *p*=1.23×10^−5^) differentiated people with type 1 diabetes. Higher serum acylcarnitines (AC) (AC C14:1, *F* value=12.25, *p*=0.001345; AC C12, *F* value=11.055, *p*=0.0018) were unique to the metabolic response to exercise in people with type 1 diabetes. C-peptide status differentially affected metabolic responses in serum ACs during exercise (AC C18:1, leverage 0.066; squared prediction error 3.07). The malic acid/pyruvate ratio in rested serum was diagnostic for maximal aerobic capacity ($\dot{V}{\text{O}}_{\text{2peak}}$) in people with type 1 diabetes (ROC curve AUC 0.867 [95% CI 0.716, 0.956]).

**Conclusions/interpretation:**

The serum metabolome distinguishes high and low maximal aerobic capacity and has diagnostic potential for facilitating personalised medicine approaches to manage aerobic exercise and fitness in type 1 diabetes.

**Graphical Abstract:**

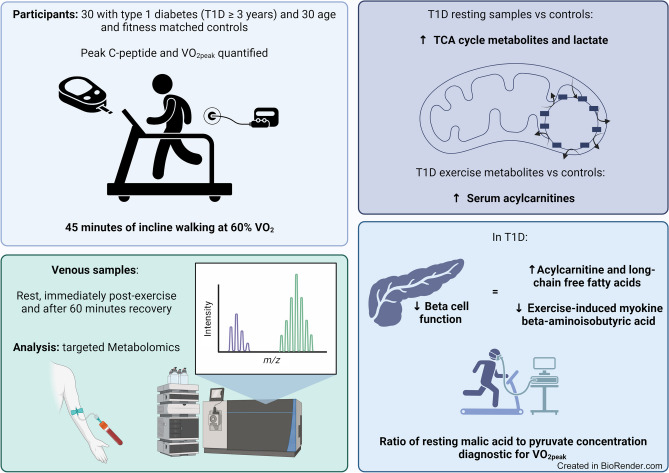

**Supplementary Information:**

The online version contains peer-reviewed but unedited supplementary material available at 10.1007/s00125-024-06153-0.


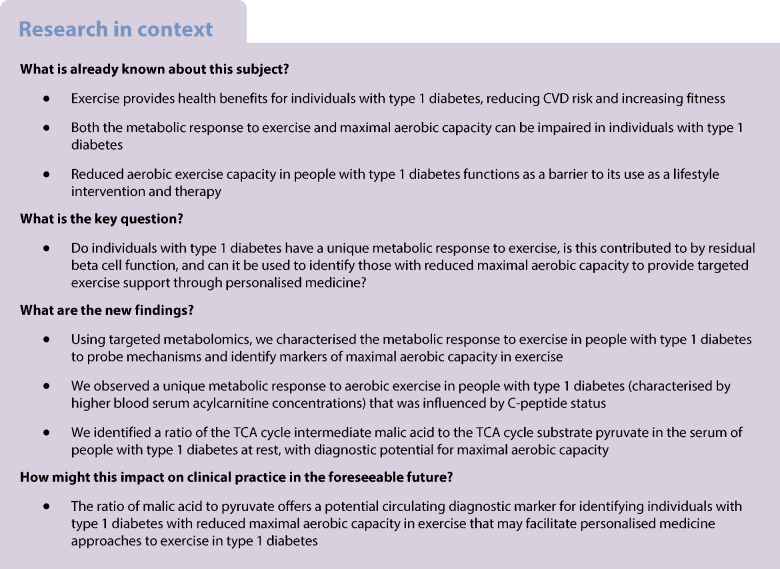


## Introduction

Exercise provides health benefits for individuals with type 1 diabetes, through reducing CVD, improving skeletal muscle health and increasing fitness [1, 2]. Despite the benefits of exercise, barriers to its use as a diabetes therapy remain. Exercise tolerance is impaired in type 1 diabetes [3–10] and the metabolic response to exercise can be acutely disrupted, with high inter-individual variability, leading to dysglycaemia [11–14]. This is despite high intra-individual reproducibility under repeated laboratory conditions, suggesting the existence of distinct subpopulations within the type 1 diabetes population exhibiting perturbed metabolic responses to exercise [15].

The variability in metabolic response to exercise in people with type 1 diabetes is not fully explained, is poorly classified and cannot be predicted [15]. A number of physiological contributors to exercise response are disrupted in people with type 1 diabetes, possibly contributing to inter-individual variation [15]. These include perturbations to fuel selection [16] in the form of carbohydrates (glycolysis) and lipids (NEFA, triacylglycerols, acylcarnitines), and anaerobic processes (lactate) and central carbon metabolism (tricarboxylic acid [TCA] cycle) [16].

The phenotype and population heterogeneity of aerobic capacity in exercise and metabolic dysfunction in type 1 diabetes makes effective screening, diagnosis and management a substantial challenge, contributing to lower activity levels within the type 1 diabetes population [16–18]. Omic approaches are a potential solution. Metabolomics has advanced understanding of metabolic diseases and is ripe for application to the heterogeneous pathological metabolic phenotype induced by exercise in people with type 1 diabetes, having become a core tool in personalised medicine [19–21]. Metabolomics is effective in defining ‘silent’ phenotypes [22] (that manifest under specific stressors) and identifying disease biomarkers preceding overt pathology [20, 21]. Therefore, metabolomics is highly appropriate for studying the metabolic perturbation in type 1 diabetes that acutely manifests under stress (increased metabolic rate, carbohydrate oxidation, and insulin sensitivity) in exercise. The study of the metabolome alongside exercise indices, such as maximal aerobic capacity ($\dot{V}{\text{O}}_{\text{2peak}}$), may provide novel information regarding an individual’s metabolic phenotype and distinguish those with lower or higher aerobic capacity.

Another unexplored mechanism possibly contributing to the metabolic response to exercise is residual beta cell function (i.e. C-peptide secretion) in people with type 1 diabetes of long duration. Between 35% and 80% of people with type 1 diabetes have detectable beta cell function at >5 years post diagnosis [23, 24], with 5–16% at the threshold for clinical benefits (peak C-peptide 200 pmol/l) found in the DCCT. As beta cell function declines in individuals with type 1 diabetes, glycaemic control deteriorates [25, 26]. Individuals with high stimulated C-peptide (>400 pmol/l) spend greater time in euglycaemia at rest compared with those with undetected, low (17–200 pmol/l) and intermediate (200–400 pmol/l) levels, suggesting an influence on basal metabolism [27]. Individuals with higher beta cell function (>200 pmol/l C-peptide) display lower glycaemic variability than individuals with low and undetectable C-peptide following aerobic exercise [28]. This suggests that residual beta cell function may contribute to variation in acute blood glucose levels following exercise in type 1 diabetes. Since C-peptide status influences metabolism (glycaemic control) post exercise, it may also contribute to the metabolic response to exercise. Application of metabolomics in people with type 1 diabetes during aerobic exercise, alongside characterisation of C-peptide status, may provide valuable insight into the impact of residual beta cell function on metabolic phenotype.

We aimed to use targeted metabolomics to characterise the metabolic signature of type 1 diabetes at rest, exercise and recovery. We explored the contribution made by residual beta cell function to metabolic response to exercise in individuals with type 1 diabetes and sought to identify circulating metabolite markers diagnostic of maximal aerobic capacity to facilitate the personalised medicine approach to diabetes.

## Methods

### Participants and recruitment

Participants with type 1 diabetes were included if they had a confirmed clinical diagnosis, age 18–65 years with type 1 diabetes duration ≥3 years, HbA_1c_ <86 mmol/mol (10%) and stable multiple daily injections or continuous insulin infusion without changes over the preceding 6 months. A diabetes duration ≥3 years avoided the ~2 year ‘honeymoon’ period [29]. Participants were excluded if they had diabetes-related complications (except non-proliferative retinopathy), other chronic conditions, history of smoking, or BP >140/90 mmHg at study visits. Participants’ clinical notes confirmed normal renal function (absence of albuminuria, GFR >60 ml/min per 1.73 m^2^). Control participants were aged 18–65 years, non-smokers and free from chronic disease, with confirmed absence of diabetes (HbA_1c_ <40 mmol/mol [5.8%]). Thirty control participants and 30 volunteers with type 1 diabetes, further subdivided into participants with either undetectable (*n*=11), low (*n*=9) or high (*n*=10) C-peptide, were recruited from Newcastle Diabetes Centre. Participant demographics are shown in Tables 1, 2 and electronic supplementary material (ESM) Table 1. The study population is representative of the larger type 1 diabetes population in terms of age and sex. Sex did not differ between control and type 1 diabetes groups and was determined by self-reporting and confirmed from participants’ clinical notes. We previously reported on this study population [28, 30]. Participants provided written informed consent prior to enrolment following approval from the NHS HRA North East Tyne & Wear South Research Ethics and Newcastle University Ethics Committees (16/NE/0192, ISRCTN63739203).

**Table 1:** Participant demographic data

Characteristic	Type 1 diabetes	Control group	*p* value
*N*	30	30	
No. male/female	16/14	16/14	
Age, years	38.2±12.0	37.6±12.1	0.84
HbA_1c_, mmol/mol	58.5±9.1	33.5±2.3	<0.001
HbA_1c_, %	7.5±3.0	5.2±2.4	<0.001
BMI, kg/m^2^	25.2±3.7	24.7±4.6	0.656
$\dot{V}{\text{O}}_{\text{2peak}}$, ml kg^−1^ min^−1^	38.8±9.5	42.4±12.4	0.205
Age at diagnosis, years	18.2±8.6	_	
Range, years	8–35		
Diabetes duration, years	20.0±13.0	_	
Range, years	3–47		
Method of control, MDI/CSII	15/15	_	
Total cholesterol, mmol/l	4.6±0.7	4.1±0.9	0.017712
Triglycerides, mmol/l	1.0±0.4	0.7±0.3	0.004876
HDL-cholesterol, mmol/l	1.6±0.4	1.6±0.3	0.897524
Total/HDL-cholesterol ratio	3.1±0.9	2.7±0.8	0.061241
Non-HDL-cholesterol, mmol/l	3.0±0.7	2.5±0.9	0.017246

Data are presented as mean ± SD

*p* value from independent samples *t* test

CSII, continuous subcutaneous insulin infusion; MDI, multiple daily injection of insulin

**Table 2:** Type 1 diabetes participant demographic data by C-peptide status and MMTT results

Characteristic	Cpep_und_	Cpep_low_	Cpep_high_	*p* value
*N*	11	9	10	
No. male / female	5/6	6/3	5/5	
Age, years	40.09±11.18	38.67±14.73	35.80±10.98	0.738
Range, years	26–58	25–61	18–53	
Age at diagnosis, years	13.27±4.5	16.56±8.57	25.1±8.2*	0.003
Range, years	8–24	8–32	13–35	
Duration of diabetes, years	26.82±13.24	21.89±13.34	10.70±6.15*	0.015
Range, years	13–47	9–44	3–20	
HbA_1c_, mmol/mol	61.64±10.64	58.11±7.11	55.40±8.47	0.297
Range, mmol/mol	42–78	51–74	41–69	
HbA_1c_, %	7.8±3.1	7.5±2.8	7.2±2.9	
Range, %	6.0–9.3	6.8–8.9	5.9–8.5	
BMI, kg/m^2^	25.65±3.27	24.20±4.13	25.67±4.04	0.259
Daily insulin dose, U	39.93±15.15	47.88±23.21	38.30±31.23	0.242
Daily insulin dose, U/kg	0.54±0.19	0.63±0.25	0.49±0.29	0.332
Method of control (MDI/CSII)	5/6	4/5	6/4	
$\dot{V}{\text{O}}_{\text{2peak}}$, ml kg^−1^ min^−1^	35.61±7.69	43.93±9.03	35.67±10.77	
Range, ml kg^−1^ min^−1^	21.05–49	31.8–58.25	21.25–51.00	0.194
Total cholesterol, mmol/l	4.5±1.1	3.7±0.7^‡^	4.0±0.7	0.0145
Triglycerides, mmol/l	0.8±0.2	0.7±0.3	0.7±0.3	0.0485
HDL-cholesterol, mmol/l	1.7±0.4	1.6±0.3	1.5±0.3	0.5876
Total/HDL-cholesterol ratio	2.8±0.9	2.5±0.7	2.8±0.8	0.2282
Non-HDL-cholesterol, mmol/l	2.8±1.1	2.2±0.7^‡^	2.5±0.7	0.0373
Peak C-peptide, pmol/l	0±0.00	42.00±32.58	671.7±435.15*^†^	<0.001
Range, pmol/l	0–0	4–83	221–1640	
Media C-peptide	0	53	568.5	

Data are presented as mean ± SD

^*^p<0.05, vs Cpep_und_; ^†^*p*<0.05 vs Cpep_low_; ^‡^*p*<0.05 vs control group (one-way ANOVA with Tukey post hoc)

Cpep_und_, undetectable C-peptide (<3 pmol/l); Cpep_high_, high C-peptide (≥200 pmol/l); Cpep_low_, low C-peptide (3–200 pmol/l); CSII, continuous subcutaneous insulin infusion; MDI, multiple daily injection of insulin

### Visit 1 mixed-meal tolerance test

Participants attended the National Institute for Health Research Newcastle Clinical Research Facility (CRF) at 08:30 hours after an overnight fast, and a cannula was inserted into an antecubital vein. Participants maintained their basal insulin regimen. Peak serum C-peptide response to a mixed-meal tolerance test (MMTT) was measured to determine residual beta cell function, according to published protocols [28]. For details, see ESM Methods. Participants were allocated into three peak C-peptide groupings: undetectable (<3 pmol/l); low (3–200 pmol/l); and high (≥200 pmol/l). For reference, peak serum C-peptide following MMTT in healthy individuals is 1000–3000 pmol/l [31, 32].

### Visit 2 maximal aerobic capacity testing

Participants’ height, weight (seca 220 stadiometer/seca 889 scale; seca, Hamburg, Germany) and medical history were recorded. Participants were screened for cardiac anomalies using a modified 12-lead resting/exercising ECG. $\dot{V}{\text{O}}_{\text{2peak}}$ and peak heart rate were defined using a maximal-graded walking treadmill (Valiant 2 CPET; Lode, Groningen, the Netherlands) test (Bruce protocol) [33]. For details, see ESM Methods.

### Visit 3 exercise intervention

The exercise protocol was conducted as previously published [28]. Participants arrived at the CRF at 08:30 hours following an overnight fast, had abstained from exercise for 48 h and maintained their basal insulin regimen. Participants were cannulated and resting (baseline) blood samples (10 ml) were drawn. Participants walked on an incline for 45 min at 60% $\dot{V}{\text{O}}_{\text{2peak}}$, then blood was drawn from the cannula immediately and after participants had rested for 60 min. Serum was separated and stored at −80°C. For details, see ESM Methods.

### Metabolomic sample preparation

An internal standard spiking solution (10 μmol/l each of palmitoyl-l-carnitine-[*N*-methyl-d3], palmitic acid-d31, deoxycholic acid-d6 and l-tryptophan-d5, and 5 µmol/l citric acid-d4; all from Sigma) in LC-MS-grade methanol was prepared. Serum (100 µl) was protein-precipitated with 400 µl internal standard spiking solution and centrifuged (3 min; 25,000 *g*). The supernatant fraction (100 µl) was diluted with 100 µl LC-MS-grade water, vortexed and transferred to LC vials. Sample order was randomised using a random number generator. Experimenters were blinded to experimental groups.

### Targeted metabolomic LC-MS analysis of acylcarnitines, NEFA, bile acids, tryptophan metabolism and TCA cycle metabolites

#### Chromatography

An Acquity UPLC system (Waters, USA) equipped with a CORTECS T3 2.7 μm (2.1×30 mm) column at 60°C was used for acylcarnitine (AC), NEFA, bile acid and tryptophan metabolite analyses and an Acquity CSH Phenyl-Hexyl 2.1×100 mm column at 80°C was used for analysis of TCA cycle intermediates, following published protocols [34]. For details, see ESM Methods.

#### MS

The Acquity UPLC system was coupled to a Xevo TQ-XS mass spectrometer (Waters). Analyses were performed using multiple reaction monitoring (MRM). For transitions and ionisation conditions, see ESM Tables 2−6. For AC and tryptophan metabolite analyses, positive electrospray ionisation was used. For NEFA, bile acid and TCA cycle analyses, negative electrospray ionisation was used. A cone gas flow rate of 50 ml/h and a desolvation temperature of 650°C were used.

#### MS data analysis

Data were processed and peak integration performed using Waters TargetLynx Version 4.1 (Waters, USA). Integrated AC, NEFA, bile acid, tryptophan metabolite and TCA cycle metabolite peak areas were normalised to the palmitoyl-l-carnitine-(*N*-methyl-d3), palmitic acid-d31, deoxycholic acid-d6, l-tryptophan-d5 or citric acid-d4 internal standard, respectively.

### Data analysis

#### Univariate

Pearson’s correlation, performed using Prism (version 6; Graphpad, USA), determined the relationship between both HbA_1c_ and metabolite concentrations and $\dot{V}{\text{O}}_{\text{2peak}}$. ANOVA, two-way repeated measures ANOVA with Bonferroni correction, and receiver operating characteristic (ROC) curve analysis were performed in MetaboAnalyst (version. 4.0.14) (https://www.metaboanalyst.ca/) [35]. Nominal significance was *p*<0.05.

#### Multivariate

Multivariate analysis was performed using MetaboAnalyst. Study size power calculations were conducted using MetaboAnalyst and published methods [36]. Datasets were mean-centred and auto-scaled before analysis using partial least-squares discriminant analysis (PLS-DA). PLS-DA models were validated using the permutation test (see ESM Methods). Metabolite changes responsible for clustering or regression trends within the pattern recognition models were identified by interrogating the corresponding loadings plot. Metabolites identified in the variable importance in projections (VIP)/coefficients plots were deemed to have changed globally if they contributed to separation in the models with 95% confidence limits and a VIP score >1. For comparisons between two groups, these were verified using univariate Student’s *t* test (fold change ≥1.2; *p*<0.05) and displayed as scatterplots or tables. For multiple group comparisons, these were confirmed by two-way ANOVA with Tukey’s or Bonferroni post hoc (as stated) and displayed as scatterplots or tables. ANOVA simultaneous component analysis (ASCA), performed in Metaboanalyst, was used for analysis of multivariate datasets, with both multiple group comparison and a longitudinal component (see ESM Methods) [37].

## Results

All participants completed the exercise protocol (mean ± SD 8.06±5.09% incline at 4.30±0.47 km/h), mean 58.8% of $\dot{V}{\text{O}}_{\text{2peak}}$, with no between-group differences (*p*=0.907). No hypoglycaemic incidences occurred during the exercise intervention. Six diabetic participants received additional carbohydrates (10 g) during exercise (when glucose <7 mmol/l): one C-peptide undetectable; two C-peptide low; and three C-peptide high. Pre- and post-exercise, and recovery blood glucose concentrations are shown in ESM Table 7 and were reported previously [28].

### HbA_1c_ concentration inversely correlates with maximal aerobic capacity in people with type 1 diabetes

**Fig. 1:**
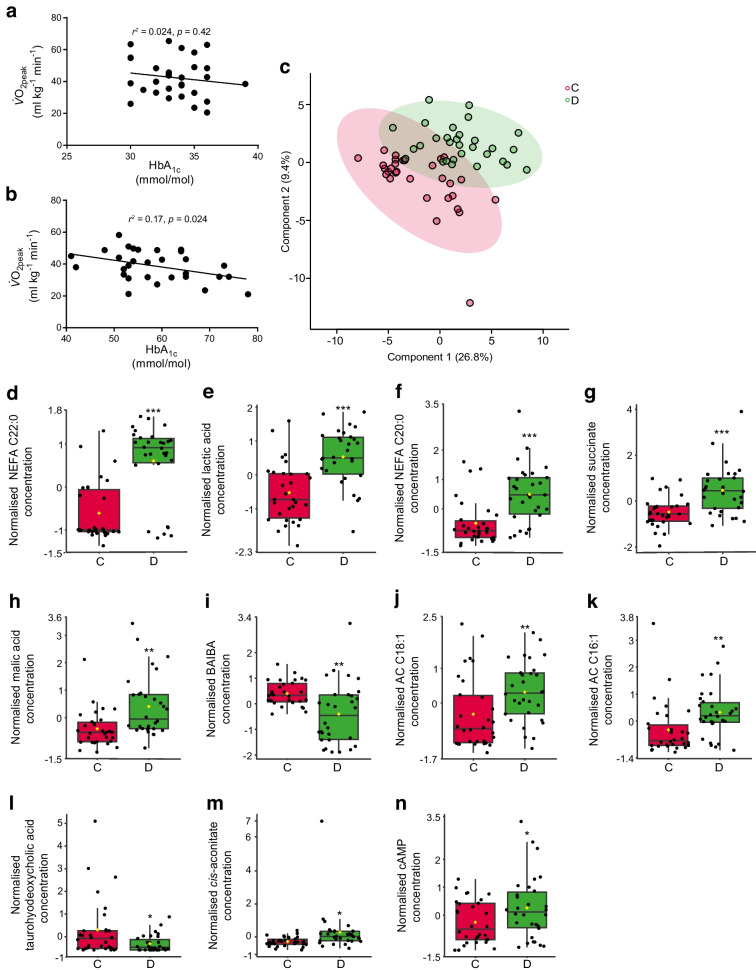
Relationship between long-term glycaemic control (HbA_1c_ mmol/mol) and maximal aerobic capacity ($\dot{V}{\text{O}}_{\text{2peak}}$) in participants with type 1 diabetes. (**a**, **b**) Correlation between HbA_1c_ and $\dot{V}{\text{O}}_{\text{2peak}}$ in non-diabetic control participants (*n*=30; *r*^*2*^=0.024, *p*=0.42) (**a**) and in participants with type 1 diabetes (*n*=30; *r*^2^=0.17, *p*=0.024) (**b**). (**c**–**n**) Metabolomics and multivariate statistics identify a metabolic signature for type 1 diabetes. (**c**) PLS-DA plot of metabolomic data from serum samples at baseline showing separation of control (red) and type 1 diabetes (green) groups. 95% confidence regions are displayed. (**d**–**n**) Metabolites distinguishing control participants from participants with type 1 diabetes at baseline as determined by PLS-DA VIP scores >1 and confirmed by Student’s *t* test. Univariate analysis is given in Table 3. Box and whisker plots show 25th and 75th percentile and the median. Upper whisker is Q3+1.5×IQR; lower whisker is Q1−1.5×IQR. Data are mean-centred and auto-scaled (normalised). Yellow point is median value. ***p<0.001, **p<0.01, *p<0.05. *n*=30 for control group and type 1 diabetes group. C, non-diabetes control; D, type 1 diabetes

The control and diabetes populations did not differ for $\dot{V}{\text{O}}_{\text{2peak}}$ (Table 1). We examined the correlation between $\dot{V}{\text{O}}_{\text{2peak}}$ and HbA_1c_ in control participants (Fig. 1a) (*r*^*2*^=0.024, *p*=0.42) and participants with type 1 diabetes using Pearson correlation. Perturbed glycaemic control (HbA_1c_) was significantly inversely correlated with $\dot{V}{\text{O}}_{\text{2peak}}$ in diabetic participants (Fig. 1b) (*r*^*2*^=0.17, *p*=0.024).

### Metabolomics identifies a metabolic signature for type 1 diabetes at baseline

To characterise the metabolic signature of people with type 1 diabetes at rest, we used targeted metabolomics to compare baseline rested serum of control participants and volunteers with type 1 diabetes. Multivariate statistics using PLS-DA identified metabolite concentrations that distinguish control participants from those with diabetes at baseline (Fig. 1c and ESM Fig. 1) (permutation test, *p*=0.0005). Differentiating metabolites were established if they both contributed to the PLS-DA model with VIP score >1 and had a significant difference between groups by univariate Student’s *t* test (Fig. 1d–n, Table 3). These included higher concentrations of long-chain NEFA, NEFA C22:0 (*p*<0.001) (Fig. 1d) and C20:0 (*p*<0.001) (Fig. 1f) and, TCA cycle intermediates succinate (*p*<0.001) (Fig. 1g), malate (*p*<0.01) (Fig. 1h) and *cis*-aconitate (*p*<0.05) (Fig. 1m), and lactate (*p*<0.001) (Fig. 1e) in the serum of diabetic participants, reflecting differential energy metabolism (Fig. 1d–n, Table 3). Serum levels of exercise-regulated myokine β-aminoisobutyric acid (BAIBA) [38, 39] were lower in diabetic participants (*p*<0.01) (Fig. 1i, Table 3).

**Table 3:** Serum metabolites significantly differing between non-diabetes control participants and participants with type 1 diabetes at rest (baseline)

Metabolite	Fold change	Log_2_(fold change)	*p* value
NEFA C22:0	1.6834	0.75142	3.63×10^−8^
Lactic acid	1.2215	0.28862	1.23×10^−5^
NEFA C20:0	1.6568	0.72838	4.45×10^−5^
Succinate	1.3117	0.39145	0.00011
Malic acid	1.3939	0.47913	0.00116
BAIBA	0.65902	−0.60161	0.001335
AC C18:1	1.5043	0.58909	0.001857
AC C16:1	1.724	0.78579	0.003675
Taurohyodeoxycholic acid	0.2695	−1.8917	0.020038
*cis*-Aconitate	1.5488	0.63113	0.025681
cAMP	1.4947	0.57985	0.044342

The table shows the fold change and log_2_(fold change) of metabolites in the serum of participants with type 1 diabetes compared with control participants at the baseline time point of the aerobic exercise study (*n*=30 for both groups)

*p* value was generated by two-tailed Student’s *t* test

### The metabolic profile of aerobic exercise in people with and without type 1 diabetes

**Fig. 2:**
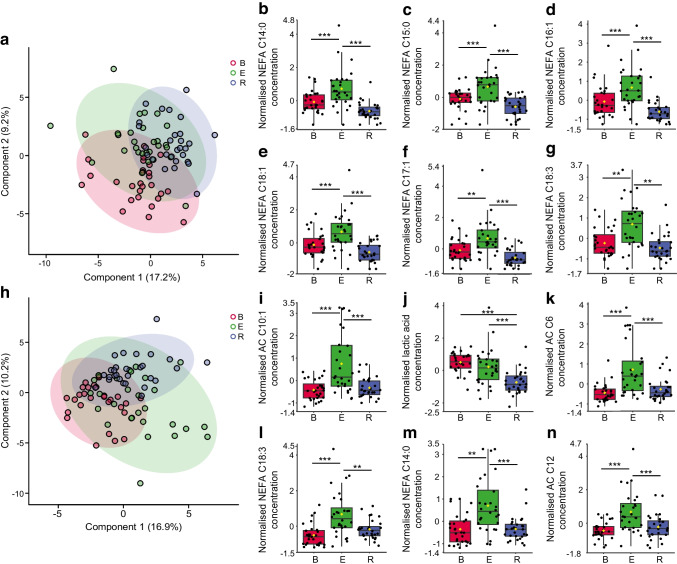
The metabolic profile of aerobic exercise in non-diabetic control participants and participants with type 1 diabetes. (**a**) Multivariate PLS-DA plot of metabolomic data from baseline (red), exercise (green) and post-exercise 1 h recovery (blue) of non-diabetic control participants identifying metabolic differences between the three states. 95% confidence regions are displayed (*n*=30). (**b**–**g**) The top six metabolites representing the metabolic profile of aerobic exercise in non-diabetic control participants (ranked by univariate *p* value; ESM Table 8) (*n*=30). (**h**) Multivariate PLS-DA plot of metabolomic data from baseline (red), exercise (green) and post-exercise 1 h recovery (blue) of people with type 1 diabetes identifying metabolic differences between the three states. 95% confidence regions are displayed (*n*=30). (**i**–**n**) The top six metabolites representing the metabolic profile of aerobic exercise in people with type 1 diabetes (ranked by univariate *p* value; Table 4) (*n*=30). Mean-centred and auto-scaled (normalised) data are shown. Box and whisker plots show 25th and 75th percentile and the median. Upper whisker is Q3+1.5×IQR, lower whisker is Q1−1.5×IQR. Yellow point is median value. ***p<0.001, **p<0.01. B, baseline; E, exercise; R, post-exercise 1 h recovery

We characterised the metabolic signature of aerobic exercise in diabetic participants. We first used targeted metabolomics of baseline, exercise and post-exercise 1 h recovery serum from control participants to build a PLS-DA model of the metabolic profile of aerobic exercise in health (Fig. 2a and ESM Fig. 2) (permutation test, *p*<0.0005). Metabolites were determined to change in response to exercise and recovery if they had a VIP score >1 and a significant difference between groups by univariate one-way ANOVA (ESM Table 8). The top six most significant metabolites ranked by univariate *p* value are shown in Fig. 2b–g. In the control participants the metabolic response to exercise was dominated by an increase in circulating long-chain NEFA, including NEFA C14:0 (*p*<0.001) (Fig. 1b), C16:1 (*p*<0.001) (Fig. 1d) and C18:1 (*p*<0.001) (Fig. 1e), which returned to baseline concentration at the recovery time point (Fig. 2b–g and ESM Table 8).

Independently, we generated a PLS-DA model to characterise the metabolic phenotype of aerobic exercise and recovery in participants with type 1 diabetes (Fig. 2h and ESM Fig. 3) (permutation test, *p*<0.0005). Metabolites were determined to change in response to exercise and recovery in diabetes if they had a VIP score >1 and a significant difference between groups by univariate one-way ANOVA (Table 4). The top six most significant metabolites ranked by univariate *p* value are shown in Fig. 2i–n. The response to exercise in diabetic participants was characterised by an increase in NEFA species, consistent with observations for control participants. However, exercise in diabetic participants was uniquely characterised by increased circulating concentrations of short-, medium- and long-chain AC species including AC C6 (*p*<0.001), AC C8 (*p*<0.01), AC C10 (*p*<0.001), AC C10:1 (*p*<0.001), AC C12 (*p*<0.001), AC C14:1 (*p*<0.01) and AC C14:2 (*p*<0.01) (Table 4).

**Table 4:** ANOVA showing serum metabolites altered following aerobic exercise in participants with type 1 diabetes

Metabolite	*F* value	*p* value	FDR	Significant group differences (Tukey’s post hoc test)
AC C10:1	16.219	1.19×10^−6^	6.61×10^−5^	E-B; E-R
Lactic acid	15.83	1.57×10^−6^	6.61×10^−5^	E-B; E-R
AC C6	14.51	4.11×10^−6^	0.000115	E-B; E-R
NEFA C18:3	13.475	8.87×10^−6^	0.000161	E-B; E-R
NEFA C14:0	13.372	9.59×10^−6^	0.000161	E-B; E-R
AC C12	12.34	2.10×10^−5^	0.000294	E-B; E-R
AC C12:1	12.045	2.63×10^−5^	0.000316	E-B; E-R
Succinate	11.746	3.32×10^−5^	0.000348	E-B; E-R
NEFA C18:1	11.365	4.46×10^−5^	0.000384	E-B; E-R
NEFA C17:1	11.335	4.57×10^−5^	0.000384	E-B; E-R
NEFA C20:4	10.868	6.59×10^−5^	0.000503	E-B; E-R
AC C14:1	9.5497	0.000189	0.001264	E-B; E-R
AC C10	9.5065	0.000196	0.001264	E-B; E-R
NEFA C16:1	9.3802	0.000217	0.0013	E-B; E-R
NEFA C16:0	8.9702	0.000303	0.001695	E-B; E-R
NEFA C15:0	8.3633	0.000499	0.00262	E-B; E-R
NEFA C16:2	8.1612	0.00059	0.002917	E-B; E-R
AC C14:2	7.8697	0.000753	0.003513	E-B; E-R
NEFA C20:3	7.6202	0.000928	0.004104	E-B; E-R
NEFA C20:5	6.578	0.002253	0.009463	E-B; E-R
AC C8	6.4956	0.002419	0.009676	E-B; E-R
NEFA C22:4	5.6039	0.005255	0.020064	E-B; E-R
Glycolithocholic acid	5.0903	0.008272	0.030209	E-B
NEFA C12:0	4.8637	0.010121	0.035424	E-B; E-R
Malic acid	4.8019	0.010696	0.035939	E-B; E-R

*N*=30

*p* value generated by one-way ANOVA

B, baseline; E, aerobic exercise; FDR, false discovery rate; R, post-exercise 1 h recovery

### The differential metabolic response to aerobic exercise in people with type 1 diabetes

**Fig. 3:**
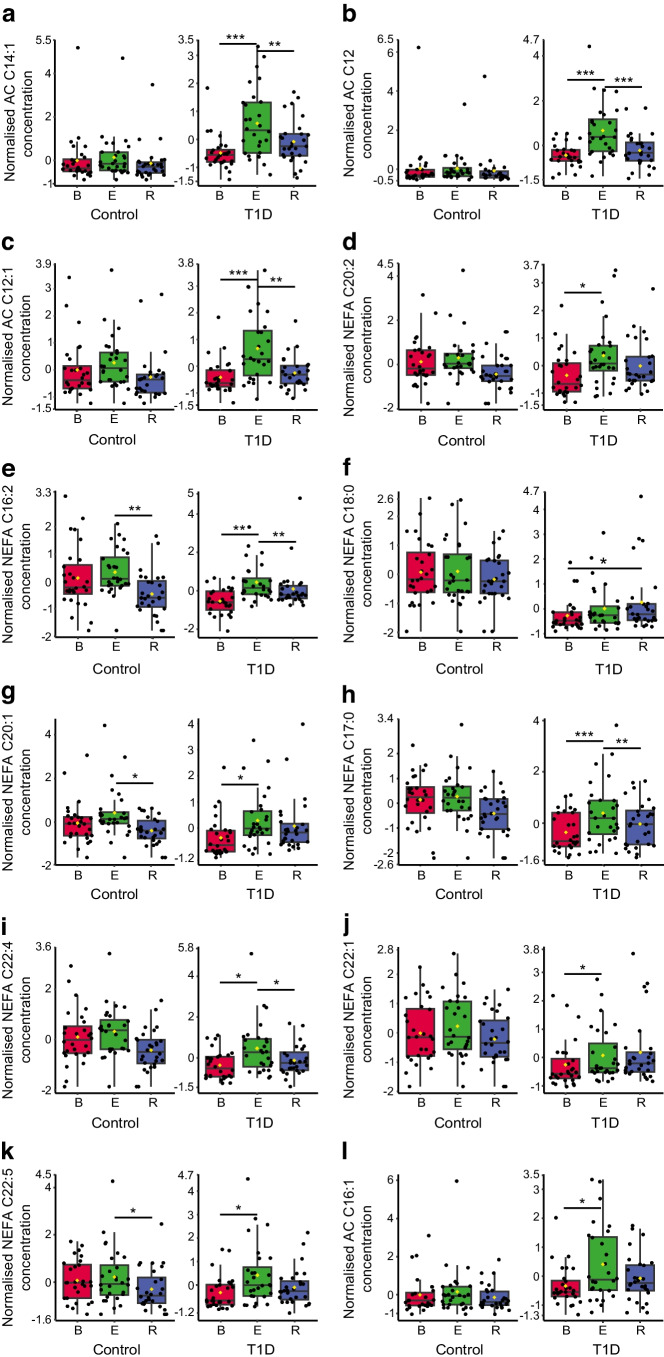
Metabolic responses to aerobic exercise distinguishing non-diabetic control participants from participants with type 1 diabetes. (**a**–**l**) Serum metabolite species from baseline (red), exercise (green) and post-exercise 1 h recovery (blue) with distinct responses to exercise in control participants and participants with type 1 diabetes, identified with MEBA for time series, which is designed to compare temporal profiles across different biological conditions. These were confirmed using corresponding univariate two-way within-subject ANOVA with Bonferroni correction (data shown in ESM Table 10). Data are mean-centred and auto-scaled (normalised). Box and whisker plots show 25th and 75th percentile and the median. Upper whisker is Q3+1.5×IQR, lower whisker is Q1−1.5×IQR. Yellow point is median value. ***p<0.001, **p<0.01, *p<0.05. Control, *n*=30; type 1 diabetes, *n*=30. B, baseline; E, exercise; R, post-exercise 1 h recovery; T1D, type 1 diabetes

We directly compared the metabolomic data for exercise in control participants vs participants with type 1 diabetes. We used both univariate (two-way repeated measures ANOVA with Bonferroni post hoc) and multivariate (multivariate empirical Bayes ANOVA [MEBA] for time series [40]) approaches to facilitate the analysis of differences between groups (diabetes, control) having two or more within-subject factors (baseline, exercise and recovery). We identified the metabolites that were commonly significant by both multivariate and univariate approaches to define the similarities (ESM Table 9, ESM Fig. 4) and differences (Fig. 3a–l and ESM Table 10) in response to exercise in control participants and those with type 1 diabetes. Common responses to exercise were observed for multiple medium-chain and long-chain NEFA (ESM Table 9; ESM Fig. 4). However, there were distinct differences in the response of several AC species, including AC C14:1 (*F* value=12.25, *p*=0.001345) (Fig. 3a), AC C12 (*F* value=11.055, *p*=0.0018) (Fig. 3b), AC C12:1 (*F* value=9.768, *p*=0.0019) (Fig. 3c) and AC C16:1 (*F* value=5.511, *p*=0.029) (Fig. 3l), which increased in response to exercise in the type 1 diabetes group only (Fig. 3 and ESM Table 10).

### A metabolic pattern distinguishes C-peptide status in people with type 1 diabetes

**Fig. 4:**
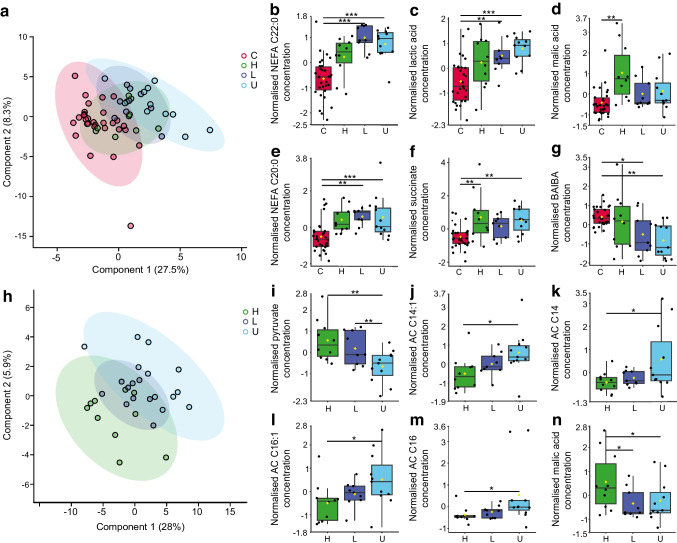
A unique metabolic pattern distinguishes C-peptide status in people with type 1 diabetes. (**a**) PLS-DA plot of metabolomic data from serum samples at baseline showing separation of control participants (red) from participants with type 1 diabetes and high (green), low (dark blue) and undetectable (light blue) plasma C-peptide. (**b**–**g**) Metabolites distinguishing control participants from diabetic participants with high, low and undetectable plasma C-peptide at baseline as determined by PLS-DA VIP scores >1 and confirmed by one-way ANOVA with Tukey’s post hoc. Univariate analysis is shown in ESM Table 11. (**h**) PLS-DA plot of metabolomic data from serum samples at baseline showing separation of high (green), low (dark blue) and undetectable (light blue) plasma C-peptide groups within the type 1 diabetes group. (**i**–**n**) Metabolites distinguishing high, low and undetectable plasma C-peptide groups at baseline as determined by PLS-DA VIP scores >1 and confirmed by one-way ANOVA with Tukey’s post hoc. Data are mean-centred and auto-scaled (normalised). Box and whisker plots show 25th and 75th percentile and the median. Upper whisker is Q3+1.5×IQR, lower whisker is Q1−1.5×IQR. Yellow point is median value. Univariate analysis is shown in ESM Table 12. ***p<0.001, **p<0.01, *p<0.05. C, control; H, high C-peptide (≥200 pmol/l); L, low C-peptide (3–200 pmol/l); U, undetectable C-peptide (<3 pmol/l)

We characterised the contribution made by residual beta cell function to the metabolic phenotype of individuals with type 1 diabetes at rest. We grouped baseline serum metabolomic data according to diabetic participants’ peak C-peptide and compared these with control participant data using PLS-DA (Fig. 4a and ESM Fig. 5) (permutation test, *p*=0.01). By examining the metabolites with both PLS-DA VIP scores >1 and significance by one-way ANOVA with Tukey’s post hoc, we identified a unique metabolic pattern associated with C-peptide status in diabetic vs control participants (Fig. 4b–g). A residual beta cell function (C-peptide concentration)-associated effect was identified in increasing serum concentrations of lactate (*p*<0.001) (Fig. 4c) and succinate (*p*<0.001) (Fig. 4f), while BAIBA (Fig. 4g) concentration decreased (*p*<0.001) with reduced C-peptide relative to control (Fig. 4b–g and ESM Table 11). Higher concentrations of long-chain NEFA C20:0 (*p*<0.01) and C22:0 (*p*<0.001) distinguished low and undetected C-peptide groups from control and high C-peptide groups (Fig. 4b,e and ESM Table 11). Group separation in PLS-DA suggested that the variation between control and C-peptide groups was greater than that between individual C-peptide groups. To explore the metabolic phenotype distinguishing the C-peptide groups we generated a PLS-DA model for the three C-peptide groups only (Fig. 4h and ESM Figure 6) (permutation test, *p*=0.0245). Interrogation of the metabolite species with VIP scores >1, with confirmed significance by one-way ANOVA with Tukey’s post hoc, identified increases in ACs C14:1 (*p*<0.05), C14 (*p*<0.05), C16:1 (*p*<0.05) and C16 (*p*<0.05) as C-peptide decreases (Fig. 4j–m and ESM Table 12). The TCA cycle substrate pyruvate (*p*<0.01) and intermediate malic acid (*p*<0.05) distinguished the metabolic phenotype of the C-peptide groups (Fig. 4i,n and ESM Table 12).

### C-peptide status influences the metabolic response to exercise in type 1 diabetes

We defined the contribution that residual beta cell function makes to the metabolic profile of exercise in individuals with type 1 diabetes. As this dataset incorporates multiple group comparison (control, undetectable, low and high C-peptide groups) across longitudinal measures (baseline, exercise, recovery), we applied ASCA, a statistical generalisation of ANOVA, to the multivariate case [37]. We identified metabolites exhibiting a differential effect to exercise dependent on C-peptide status (ESM Table 13, ESM Fig. 7). Distinguishing metabolites included AC C16:1 (leverage=0.05; squared prediction error=5.94), AC C18:1 (leverage=0.066; squared prediction error=3.07), NEFA C20:0 (leverage=0.064; squared prediction error=4.45) and BAIBA (leverage=0.062; squared prediction error=8.14).

### The basal metabolome is associated with maximal aerobic capacity in people with type 1 diabetes

We used Pearson correlation to establish the metabolites from the baseline serum samples that significantly correlated with $\dot{V}{\text{O}}_{\text{2peak}}$ across our total study population (ESM Table 14). Multiple serum long-chain NEFAs demonstrated a significant inverse correlation to $\dot{V}{\text{O}}_{\text{2peak}}$.

We then identified the relationship between the baseline serum metabolome and $\dot{V}{\text{O}}_{\text{2peak}}$ specifically relevant to our type 1 diabetes and control groups to investigate whether distinct metabolites associated with maximal aerobic capacity in type 1 diabetes. The long-chain NEFAs were significantly inversely correlated with $\dot{V}{\text{O}}_{\text{2peak}}$ in our control group (ESM Table 15). Contrastingly, serum NEFAs did not significantly correlate with $\dot{V}{\text{O}}_{\text{2peak}}$ in type 1 diabetes (Table 5). The TCA cycle intermediates malic acid (*r*=−0.5, *p*<0.01) and *cis*-aconitate (*r*=−0.4, *p*<0.05) inversely correlated with $\dot{V}{\text{O}}_{\text{2peak}}$ in diabetic participants, while the TCA cycle substrate pyruvate (*r*=0.4, *p*<0.05) significantly positively correlated with $\dot{V}{\text{O}}_{\text{2peak}}$ (Table 5).

**Table 5:** Baseline rested serum metabolites significantly correlated with $\dot{V}{\text{O}}_{\text{2peak}}$ in the type 1 diabetes study population

Metabolites	Correlation	*t* statistic	*p* value
Malic acid	−0.49834	−3.0415	0.005068
NEFA C12:0	−0.48092	−2.9025	0.00714
*cis*-Aconitate	−0.39626	−2.2837	0.030175
Pyruvate	0.36872	2.099	0.044959

Correlation analysis by Pearson *r* showing correlation, *t* statistic and *p* value; *N*=30

### The metabolome as a diagnostic for maximal aerobic capacity in people with type 1 diabetes

To further investigate the potential of the metabolome as a diagnostic tool in determining lower maximal aerobic capacity in people with type 1 diabetes, we structured our dataset for diagnostic analysis by separating the metabolomic data for control and type 1 diabetes populations. These were classified as either low maximal aerobic capacity (bottom 50% of the study population for $\dot{V}{\text{O}}_{\text{2peak}}$; positive for condition) or high maximal aerobic capacity (top 50% of the study population for $\dot{V}{\text{O}}_{\text{2peak}}$; negative for condition). We used ROC curves to investigate whether metabolites have diagnostic potential in distinguishing individuals with lower maximal aerobic capacity from those with higher maximal aerobic capacity within both the control and type 1 diabetes population. ROC curves plot the sensitivity against specificity of a test [41]. The AUC summarises the test diagnostic efficacy: 0 indicates a wholly inaccurate test; 1 indicates a fully accurate test; and 0.7–0.8 and 0.8–0.9 indicates acceptable and excellent diagnostic efficacy, respectively [41].

**Fig. 5:**
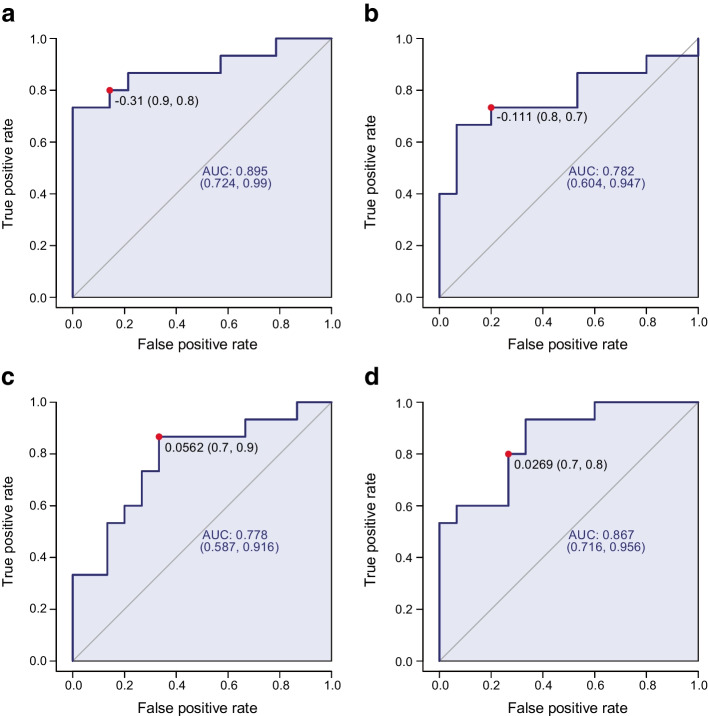
ROC curve diagnostic assessment of maximal aerobic capacity (distinguishing top and bottom 50% of population for $\dot{V}{\text{O}}_{\text{2peak}}$) for NEFA 16:1 in baseline serum of healthy control participants (*p*=0.0026) (**a**), and pyruvate (*p*=0.013) (**b**), malic acid (*p*=0.013) (**c**) and the malic acid/pyruvate ratio (*p*=0.00018) (**d**) in baseline serum of participants with type 1 diabetes. ROC curves indicate cut offs, sensitivity, specificity, AUC and 95% CI. The diagonal line represents points at which the false positive rates = the true positive rates. The red dot indicates the optimal cutoff with the associated sensitivity and specificity values. Control participants, *n*=30; participants with type 1 diabetes, *n*=30

ROC curve analysis of baseline serum metabolites of control participants with low and high maximal aerobic capacity indicated that NEFA C16:1 had the greatest ROC curve AUC (0.895 [95% CI 0.724, 0.99]; *p*=0.0026, sensitivity=0.9, specificity=0.8) (Fig. 5a). Rested serum NEFA C16:1 exhibited excellent diagnostic efficacy in differentiating low from high maximal aerobic capacity in healthy individuals.

Next, we determined the metabolome’s utility in distinguishing people with low vs high maximal aerobic capacity in our type 1 diabetes population. ROC curve analysis of baseline serum metabolites in low and high maximal aerobic capacity diabetic participants identified that the TCA cycle substrate pyruvate was the most discriminatory metabolite (AUC 0.782 [95% CI 0.604, 0.947]; *p*=0.013, sensitivity=0.8, specificity=0.7) (Fig. 5b), followed by the TCA cycle metabolite malic acid (AUC 0.778 [95% CI 0.587, 0.916]; *p*=0.013, sensitivity=0.7, specificity=0.9) (Fig. 5c), with AUCs of 0.7–0.8 indicating these are only adequate as diagnostic indicators of maximal aerobic capacity.

Serum pyruvate and malic acid exhibited reciprocal correlations with maximal aerobic capacity in the type 1 diabetes population: pyruvate was positively and malic acid negatively correlated to $\dot{V}{\text{O}}_{\text{2peak}}$ (Table 5). Therefore, we examined whether the malic acid/pyruvate ratio is a more specific and sensitive diagnostic marker for maximal aerobic capacity in the type 1 diabetes population. ROC curve analysis identified that the malic acid/pyruvate ratio (AUC 0.867 [95% CI 0.716, 0.956]; *p*=0.00018, sensitivity=0.7, specificity=0.8) (Fig. 5d), displayed a stronger diagnostic efficiency than either of the metabolites alone, suggesting that this ratio offers potential as a diagnostic marker for maximal aerobic capacity in individuals with type 1 diabetes.

## Discussion

We used targeted metabolomics to define the metabolic baseline phenotype and response to exercise in serum of individuals with type 1 diabetes and control individuals. We showed that residual beta cell function contributes to the metabolic phenotype of individuals with type 1 diabetes both at rest and in response to acute aerobic exercise. During aerobic exercise, NEFAs are released from triacylglycerol in adipose tissue via lipolysis and enter the circulation to provide fuel for contracting muscle, where they are converted to ACs to enter the mitochondria and undergo β-oxidation [42–44]. This response is reflected in our data by the increased serum NEFAs common to control and diabetic participants in aerobic exercise. Unique to people with type 1 diabetes was an increase in serum ACs in aerobic exercise, a feature that also distinguishes the metabolic effect of lower residual beta cell function. We identified that the serum metabolome of both control and diabetic participants at rest was related to maximal aerobic capacity. In healthy volunteers, this relationship was characterised by an inverse correlation between medium/long-chain NEFAs and $\dot{V}{\text{O}}_{\text{2peak}}$. In diabetic participants this relationship was distinctly described by an inverse correlation with TCA cycle metabolites malic acid and *cis*-aconitate and a positive correlation with TCA cycle substrate pyruvate. A key driver for reduced physical activity in people with type 1 diabetes is fear of hypoglycaemia [18], although this is multifactorial, with exercise intolerance [3–10] and high inter-individual variation in the metabolic response to exercise [16, 17] contributing. Such barriers limit the use of exercise as a therapy and reduce population-wide uptake, with low maximal aerobic capacity contributing to worse outcomes and risk of diabetes complications [15]. Therefore, we used ROC curves to identify the diagnostic potential of metabolites for maximal aerobic capacity. The malic acid/pyruvate ratio was revealed to be a putative diagnostic marker for differentiating maximal aerobic capacity specifically in individuals with type 1 diabetes. Recently, altered mitochondrial function has been described in the skeletal muscle in type 1 diabetes [2, 45, 46]. Moreover, blood ACs are a marker of mitochondrial (dys)function [44, 47]. Therefore, perturbed serum ACs and TCA cycle intermediates, mitochondrial metabolite species, during exercise may reflect dysfunction of skeletal muscle mitochondria in type 1 diabetes.

Study limitations include participant recruitment from a single cohort and centre. Most of the diabetic participants had good glycaemic control and exercise testing was conducted under laboratory conditions with acute exercise intervention and close monitoring of blood glucose levels. Although our experimental groups did not differ for sex and therefore our findings may be generalisable across sexes in the type 1 diabetes population, we did not directly investigate interacting variables (e.g. age, sex and duration of diabetes, which were not significantly different between groups in our study) that may contribute to both the metabolome and maximal aerobic capacity in the diabetic population.

Exogenous insulin may blunt the metabolic response to exercise in type 1 diabetes [48]. We did not observe this phenomenon, possibly because our volunteers with type 1 diabetes arrived fasted, maintaining only basal exogenous insulin delivery. In addition, although C-peptide is a robust equimolar insulin secretion marker and indicator of beta cell function in response to an MMTT [49], other factors such as insulin resistance and renal impairment can affect serum C-peptide concentration [50]. However, these increase C-peptide concentrations [50] and given our volunteers have normal kidney function and did not exhibit between-group difference for HbA_1c_, are unlikely to have affected our study. Additionally, C-peptide has direct insulin-independent metabolic effects on several tissues (e.g. sensory nerves, vasculature) [31]. The C-peptide-associated metabolic responses to exercise may be influenced by these direct effects and not residual beta cell insulin secretion alone. This may be pertinent, as the high-C-peptide group had a slightly shorter duration of diabetes than comparator groups. Nevertheless, four volunteers within this group had diabetes for >15 years, and recruitment of volunteers ≥3 years post diagnoses reduces the impact of the early honeymoon phase.

To our knowledge, we are the first to suggest a circulating metabolome-derived marker of maximal aerobic capacity in people with type 1 diabetes. This approach may facilitate future studies validating the serum malic acid/pyruvate ratio as a biomarker of maximal aerobic capacity in the wider type 1 diabetes population. Our approach may expedite identification of patients with type 1 diabetes and lower maximal aerobic capacity to target structured support for exercise. Moreover, future, higher-powered, studies will be important to investigate the influence of other interacting variables (e.g. age of diabetes onset, diabetes duration) and could focus on whether metabolomic signatures associate with increased hypoglycaemic risk during and post exercise. This approach could enable the identification of individuals at highest risk and generate personalised medicine strategies to reduce exercise-induced hypoglycaemia in those most in need. It will be important to establish the metabolomic response to exercise in fasting, fed and high and low exogenous insulin conditions, while carefully avoiding hypo- and hyperglycaemia, to determine the contribution of exogenous insulin to the metabolic response to aerobic exercise in type 1 diabetes. Studies of the interaction between the metabolome and exercise capacity in differing exercise modalities such as resistance and high-intensity interval training, with physiological markers distinct from $\dot{V}{\text{O}}_{\text{2peak}}$ and over longer durations, are needed. The analysis of blood ACs and TCA cycle intermediates in people with type 1 diabetes, with concomitant assessment of muscle mitochondrial function, may inform personalised medicine approaches to target aerobic exercise programmes to improve muscle health.

In summary, people with type 1 diabetes exhibit a unique metabolic phenotype in response to aerobic exercise, characterised by increased circulating ACs. We identify a metabolic signature of perturbed serum TCA cycle intermediates at rest, correlating with maximal aerobic capacity in people with type 1 diabetes. The serum malic acid/pyruvate ratio may have diagnostic potential in determining individuals with type 1 diabetes and lower maximal aerobic capacity. The putative metabolite markers may inform identification, management and therapy of lower maximal aerobic capacity in exercise in people with type 1 diabetes.

### Supplementary Information

Below is the link to the electronic supplementary material.

ESM1 (PDF 1504 KB)

## Data Availability

All data are available from the authors on reasonable request.

## Abbreviations

AC: Acylcarnitine
ASCA: ANOVA simultaneous component analysis
BAIBA: β-Aminoisobutyric acid
CRF: Clinical research facility
MEBA: Multivariate Empirical Bayes ANOVA
MMTT: Mixed-meal tolerance test
PLS-DA: Partial least-squares discriminant analysis
ROC: Receiver operating characteristic
TCA: Tricarboxylic acid
VIP: Variable importance in projections
