# Supplementary material for: The metabolome as a diagnostic for maximal aerobic capacity during exercise in type 1 diabetes
Source: Diabetologia. 2024 Apr 25;67(7):1413–28. doi: 10.1007/s00125-024-06153-0 (PMC11153288; doi:10.1007/s00125-024-06153-0)
Supplement: Supplementary file 1 — ESM1 (PDF 1504 KB) [file 125_2024_6153_MOESM1_ESM.pdf]

Electronic Supplemental Material (ESM): The metabolome as a diagnostic for maximal aerobic capacity during exercise in type 1 diabetes

Short title: The exercise capacity metabolome in diabetes

Guy S. Taylor[1], Kieran Smith[1,2], Jadine Scragg[1,3] Timothy J. McDonald[4], James A. Shaw[5], Daniel J. West[1] and Lee D. Roberts[6]

1. Human Nutrition & Exercise Research Centre, Population Health Sciences Institute, Newcastle University, Newcastle upon Tyne, U.K.

2. The Oxford Centre for Diabetes, Endocrinology and Metabolism, Churchill Hospital, University of Oxford, Oxford, UK

3. Nuffield Department of Primary Care Health Sciences, University of Oxford, Oxford, UK

4. University of Exeter Medical School, University of Exeter, Exeter, UK

5. Translational and Clinical Research Institute, Newcastle University, Newcastle upon Tyne, U.K

6. Leeds Institute of Cardiovascular and Metabolic Medicine, University of Leeds, Leeds, UK.

ESM Research Design and Methods

Visit 1 – Mixed Meal Tolerance Test

The measurement of peak serum C-peptide in response to a mixed meal tolerance test is the recommended assessment of residual beta-cell function by the Type 1 Diabetes TrialNet Research Group, the European C-peptide Trial (ECPT) Study Group and the America Diabetes Association [1, 2]. Participants received 240 mL Fortisip (Nutricia, Trowbridge, U.K.) (1506.24 kJ [360 kcal], 14.4 g protein, 13.92 g fat, and 44.16 g carbohydrate) over 2 min [1]. Blood was sampled at baseline and every 30 min up to 180 min. Samples were centrifuged and plasma and serum was stored at -80°C in the Newcastle Biobank facility. Potential participants with >3 years' type 1 diabetes duration were stratified by peak C-peptide status. Participants with type 1 diabetes were stratified by peak C-peptide status using an electrochemiluminescence immunoassay (E170 analyzer; Roche) of plasma following the mixed meal tolerance test. C-peptide analysis was performed at the Exeter Clinical Laboratories, University of Exeter.

Visit 2 – Maximal Aerobic Capacity Testing

Participants wore a face mask (Hans Rudolph 7450 Series V2 mask) to collect expired air which was immediately measured by an online gas analyser using indirect calorimetry (Metalyzer® 3B-R3 CPET, Cortex, Germany). The test was considered a maximal effort if participants met two of the following criteria: a distinct plateau in oxygen consumption, a peak hr ≥ 90% age predicted maximum HR (220-age), a rating of perceived exertion (RPE) of ≥ 18 and / or respiratory exchange ratio (RER) of 1.15 or greater. The peak rate of oxygen uptake and utilization during the maximal intensive exercise (VO_2Peak) was determined by averaging the highest consecutive 30 seconds VO_2 value before voluntary exhaustion.

The highest number of beats per minute recorded during maximal exercise test was determined as maximal heart rate (HRpeak).

Visit 3 Exercise Intervention

Individuals arrived at the CRF exercise laboratory at 8:30 A.M. following an overnight fast. Participants maintained their normal basal insulin regimen and had abstained from exercise for 48 hr. A carbohydrate snack (belVita; Mondelez International) of 853.54 kJ (204 kcal; 31 g carbohydrate) was consumed, and participants were rested for 20 min.

Target capillary blood glucose was > 7 mmol/L during exercise, with participants given 10 g carbohydrate if capillary blood glucose decreased below this limit.

Each participant was cannulated and resting (baseline) blood samples (10 mL) were drawn. HbA1c was measured in baseline blood. Participants walked on an incline for 45 minutes at 60% $\dot{V}O_2Peak$. Participants' treadmill velocity and gradient were calculated using $\dot{V}O_2$, velocity, and gradient data from the preliminary $\dot{V}O_2Peak$ test. Breath-by-breath respiratory parameters (Metalyzer 3B-R3, Cortex) were recorded, with gradient adjusted at 10 and 30 minutes if $\dot{V}O_2$ was >10% different than target $\dot{V}O_2$. Upon exercise completion, blood samples were drawn from the cannula. Participants rested for 60 minutes before final blood samples were drawn. At each time point whole EDTA venous blood glucose was analysed using a YSI 2300 STAT PLUS Analyzer (YSI Inc, Xylem Analytics, USA). Blood was processed for serum and it was stored at - 80°C.

Targeted Metabolomics LC−MS Analysis of Acylcarnitines, Non-esterified fatty acids, Bile Acids, Tryptophan Metabolism and TCA Cycle Metabolites - Chromatography

The binary solvent system used for the analysis of acylcarnitines, non-esterified fatty acids, bile acids and tryptophan metabolites was solvent A comprising LC−MS-grade water, 0.2 mM ammonium formate, and 0.01% formic acid and solvent B comprising analytical-grade acetonitrile/isopropanol 1:1, 0.2 mM ammonium formate, and 0.01% formic acid. For the analysis of acylcarnitines, non-esterified fatty acids and bile acids the mobile phase was set

at a flow rate of 1.3 mL/min. For the tryptophan metabolite analysis the mobile phase was set at a flow rate of 0.45 mL/min.

For acylcarnitine analysis, the column mobile phase was held at 2% solvent B for 0.1 min, followed by an increase from 2 to 98% solvent B over 1.2 min. The mobile phase was then held at 98% solvent B for 0.9 min. The mobile phase was then returned to 2% solvent B and held for 0.1 min to re-equilibrate the column. For the analyses of acylcarnitines 0.5 μL of sample was injected.

For non-esterified fatty acid analysis, the column mobile phase was increased from 50 to 98% solvent B over 0.7 min. The mobile phase was then held at 98% solvent B for 0.5 min. The mobile phase was then returned to 50% solvent B and held for 0.1 min to re-equilibrate the column. For the analyses of free fatty acids 2 μL of sample was injected.

For bile acid analysis, the column mobile phase was held at 20% solvent B for 0.1 min, followed by an increase from 20 to 55% solvent B over 0.7 min. The mobile phase was increased to 98% solvent B and held for 0.9 min. The mobile phase was then returned to 20% solvent B held for 0.1 min to re-equilibrate the column. For the analyses of bile acids 10 μL of sample was injected.

The binary solvent system used for the analysis of TCA cycle metabolites was solvent A comprising LC−MS-grade water and 0.1% formic acid and solvent B comprising LC-MS-grade acetonitrile and 0.1% formic acid. The mobile phase was set at a flow rate of 0.4 mL/min. The column mobile phase was increased from 2% solvent B to 15% solvent B over 3 min. The mobile phase was then returned to 2% solvent B and held for 0.1 min to re-equilibrate the column. Three μL of sample was injected.

Data Analysis

Multivariate – PLS-DA Permutation Test

In the Permutation Test the class labels for the dataset are randomly reassigned and a new classifier is built using the original data, its performance is then evaluated. This process is repeated with 2000 permutations to estimate the distribution of the group separation distance[3]. By comparing the performance of the original model and the performance of the 2000 randomly assigned classifiers, it can be determined whether the original model is significantly different from the 2000 random permutations. The further to the right of the distribution formed by randomly permuted data in the permutation test statistic, the more significant the discrimination. The *P*-value is calculated from the proportion of times that class separation based on the randomly labelled sample is as good as the original data.

ANOVA Simultaneous Component Analysis (ASCA)

ASCA is a direct generalisation of ANOVA for univariate data to the multivariate setting, suitable for the analysis of datasets that incorporate both a group comparison and longitudinal structure. The results of this analysis can be visualised with a plot of leverage against the squared prediction error (SPE). Leverage measures the influence of each observation for a principal component. Score plots will identify observations with high leverages, ie. observations that tend to contribute mostly to separation in the PCA. SPE measures the expected squared distance between the predicted value and the true value (i.e. the quality of the predictor).

ESM Tables

**Pharmacological Agent**	**Control** (N = 30)	**$Cpep_{und}$** (N = 11)	**$Cpep_{low}$** (N = 9)	**$Cpep_{high}$** (N = 10)
Metformin use		1	1	
Budesonide/formoterol inhaler use				1
Atorvastatin use		1	1	1
Simvastatin use		1		
Sertraline use	1		1	1
Lercanidipine use		1		

ESM Table 1. Volunteer pharmacological treatments by group.

Acylcarnitine	Parent Ion (*m/z*)	Fragment Ion (*m/z*)	Cone Voltage (v)	Collision Energy (ev)
C18:2	424.3	85	50	28
C18:1	426.4	85	50	28
C18	428.4	85	50	28
C16:1	398.3	85	50	26
C16	400.3	85	50	26
C14:2	368.3	85	46	26
C14:1	370.3	85	46	26
C14	372.3	85	46	26
C12:1	343.3	85	46	24
C12	344.3	85	46	24
C10:1	314.2	85	42	24
C10	316.2	85	42	24
C8:1	286.2	85	42	22
C8	288.2	85	42	22
C6	260.2	85	54	20
C5:1	244.2	85	38	22
C5	246.1	85	38	22
C4	232.1	85	34	20
C3	218.1	85	32	18
C2	204.1	85	32	18
C16:0-d3 IS	403.4	341.26	8	18

ESM Table 2. Multiple Reaction Monitoring Parameters for acylcarnitines species. Acylcarnitines are designated by acyl chain length in carbons and degree of unsaturated double bonds. Internal standard (IS). *m/z* mass-to-charge ratio.

Non-esterified Fatty Acid	Parent Ion (*m/z*)	Fragment Ion (*m/z*)	Cone Voltage (v)	Collision Energy (ev)
C22:6	327.25	327.25	45	7
C22:5	329.25	329.25	45	7
C22:4	331.25	331.25	45	7
C22:1	337.25	337.25	45	7
C22:0	339.25	339.25	45	7
C20:5	301.25	301.25	45	7
C20:4	303.25	303.25	45	7
C20:3	305.25	305.25	45	7
C20:2	307.25	307.25	45	7
C20:1	309.25	309.25	45	7
C20:0	311.25	311.25	45	7
C18:3	277.25	277.25	45	7
C18:2	279.25	279.25	45	7
C18:1	281.25	281.25	45	7
C18:0	283.25	283.25	45	7
C17:1	267.25	267.25	45	7
C17:0	269.25	269.25	45	7
C16:2	251.25	251.25	45	7
C16:1	253.25	253.25	45	7
C16:0	255.25	255.25	45	7
C15:1	239.25	239.25	45	7
C15:0	241.25	241.25	45	7
C14:1	225.25	225.25	45	7
C14:0	227.25	227.25	45	7
C12:1	197.25	197.25	45	7
C12:0	199.25	199.25	45	7
C16:0-d31 IS	286.62	286.62	45	7

ESM Table 3. Multiple Reaction Monitoring Parameters for Non-esterified fatty acid species. Non-esterified fatty acids are designated by acyl chain length in carbons and degree of unsaturated double bonds. Internal standard (IS). *m/z* mass-to-charge ratio.

**Bile Acid**	**Parent Ion (*m/z*)**	**Fragment Ion (*m/z*)**	**Cone Voltage (v)**	**Collision Energy (ev)**
Glycoursodeoxycholic acid	448.25	74	60	35
Tauroursodeoxycholic acid	498.25	80	60	60
Taurohyodeoxycholic acid	498.25	80	60	60
Taurocholic acid	514.25	80	60	64
Glycocholic acid	464.25	74	60	34
Taurochenodeoxycholic acid	498.25	80	60	60
Taruodeoxycholic acid	498.25	80	60	60
Ursodeoxycholic acid	391.25	391.25	60	16
Cholic acid	407.25	343.25	60	34
Glycochenodeoxycholic acid	448.25	74	60	35
Glycodeoxycholic acid	448.25	74	60	35
Taurolithocholic acid	482.25	80	60	60
Chenodeoxycholic acid	391.25	391.25	60	16
Glycolithocholic acid	432.25	74	60	35
Deoxycholic acid	391.25	391.25	60	16
Lithocholic acid	375.2	373.25	60	32
Deoxycholic acid-d6 IS	397.23	331.32	80	36

ESM Table 4. Multiple Reaction Monitoring Parameters for bile acid species. Internal standard (IS). *m/z* mass-to-charge ratio.

**Tryptophan Metabolite**	**Parent Ion (*m/z*)**	**Fragment Ion (*m/z*)**	**Cone Voltage (v)**	**Collision Energy (ev)**
Tryptophan	205.17	118.11	20	24
Kynurenic acid	190.14<br>190.14<br>190.14	89.08<br>116.11<br>144.12	40<br>40<br>40	34<br>30<br>15
Kynurenine	209.17<br>209.17	94.09<br>192.14	20<br>20	12<br>6
Tryptamine	161.07	144.01	20	14
Serotonin	177.13<br>177.13	115.10<br>160.13	30<br>30	24<br>9
Melatonin	233.15	174.12	4	13
5-HIAA	192.07	146.04	10	13
Acetyl-5-hydroxy-tryptamine	219.07	160.17	35	8

ESM Table 5. Multiple Reaction Monitoring Parameters for tryptophan metabolites. *m/z* mass-to-charge ratio.

**TCA Cycle Intermediates**	**Parent Ion (*m/z*)**	**Fragment Ion (*m/z*)**	**Cone Voltage (v)**	**Collision Energy (ev)**
Lactic acid	88.97	42.96	22	8
Malic acid	133.1<br>133.1	114.91<br>71.00	34<br>34	10<br>10
Succinic acid	117.05	73.00	22	12
Isocitric acid	191.1	155	20	12
Citric acid	191.1	110.94	16	10
Fumaric acid	115.1<br>115.1	41.00<br>70.94	32<br>32	6<br>6
Pyruvic acid	87.16	42.96	28	6
α-Ketoglutarate	145.1	100.91	22	6
Phosphoenolpyruvate	166.6	79.00	28	6
Cis-Aconitic acid	172.97	128.9	22	8

ESM Table 6. Multiple Reaction Monitoring Parameters for TCA cycle intermediates.

m/z mass-to-charge ratio.

	Type 1 diabetes	Controls	*p*-value
**Pre-exercise blood glucose (mmol/L)**	7.87 ± 2.16	4.36 ± 0.38	<0.0001
**Post-exercise blood glucose (mmol/L)**	10.12 ± 3.72	4.93 ± 0.36	<0.0001
**Post-exercise 1 hr recovery blood glucose (mmol/L)**	11.15 ± 3.47	4.32 ± 0.32	<0.0001

ESM Table 7. Type 1 diabetes and control participants blood glucose results pre-exercise, immediately post-exercise and after 1hr recovery post-exercise. Data presented as mean ± SD.

Metabolite	F-value	*p*-value	FDR	Significant Group Differences (Tukey's Post-hoc test)
NEFA C14:0	19.053	1.65×10^{-7}	1.39×10^{-5}	E-B; R-E
NEFA C15:0	15.667	1.77×10^{-6}	4.54×10^{-5}	E-B; R-E
NEFA C16:1	15.463	2.05×10^{-6}	4.54×10^{-5}	E-B; R-E
NEFA C18:1	15.311	2.29×10^{-6}	4.54×10^{-5}	E-B; R-E
NEFA C18:3	14.878	3.14×10^{-6}	4.54×10^{-5}	E-B; R-E
NEFA C17:1	14.834	3.24×10^{-6}	4.54×10^{-5}	E-B; R-E
NEFA C16:0	13.631	7.89×10^{-6}	9.47×10^{-5}	E-B; R-B; R-E
5-HIAA	13.155	1.13×10^{-5}	0.000119	E-B; R-E
lactic acid	12.795	1.48×10^{-5}	0.000138	E-B; R-B
NEFA C12:0	11.823	3.13×10^{-5}	0.000263	E-B; R-E
Succinate	10.797	6.97×10^{-5}	0.000532	E-B; R-E
Glycolithocholic acid	7.8958	0.000737	0.005156	E-B; R-E
NEFA C20:3	7.7959	0.000801	0.005175	R-E
Kynurenic acid	6.8582	0.001772	0.010631	E-B; R-E
NEFA C20:4	6.0818	0.003461	0.01938	R-E
NEFA C15:1	5.6438	0.005074	0.026637	E-B
NEFA C16:2	5.2509	0.007174	0.035448	R-E
NEFA C20:1	5.1194	0.008061	0.037616	R-E
NEFA C20:5	4.7373	0.011332	0.048721	R-E
taurochenodeoxycholic acid	4.7112	0.0116	0.048721	E-B

ESM Table 8. ANOVA showing serum metabolites altered following aerobic exercise in non-diabetes controls. NEFA = Non-esterified Fatty Acid. FDR = False Discovery Rate. P – value generated by One-Way ANOVA. Significant between group differences calculated using a Tukey's Post-hoc test. Group B = baseline, group E = exercise, group R = post exercise 1 hour recovery. N = 30.

**Metabolite**	**Exercise (F.val)**	**Exercise (raw *p*-value)**	**Exercise (adj. *p*-value)**
NEFA C14:0	51.244	2.24×10^{-16}	1.88×10^{-14}
NEFA C18:3	49.088	6.86×10^{-16}	2.88×10^{-14}
NEFA C18:1	46.876	2.21×10^{-15}	6.19×10^{-14}
lactic acid	45.903	3.73×10^{-15}	7.00×10^{-14}
NEFA C17:1	45.7	4.17×10^{-15}	7.00×10^{-14}
NEFA C15:0	40.78	6.41×10^{-14}	7.08×10^{-13}
NEFA C16:1	41.118	5.28×10^{-14}	7.08×10^{-13}
NEFA C16:0	40.691	6.74×10^{-14}	7.08×10^{-13}
Succinate	33.586	4.55×10^{-12}	4.24×10^{-11}
NEFA C12:0	30.441	3.28×10^{-11}	2.75×10^{-10}
NEFA C20:3	29.98	4.40×10^{-11}	3.36×10^{-10}
NEFA C20:4	28.724	9.93×10^{-11}	6.42×10^{-10}
5-HIAA	28.236	1.37×10^{-10}	8.21×10^{-10}
Kynurenic acid	26.501	4.32×10^{-10}	2.42×10^{-9}
NEFA C20:5	24.471	1.72×10^{-9}	9.02×10^{-9}
AC C10:1	21.854	1.07×10^{-8}	5.00×10^{-8}

ESM Table 9. Two-way repeated measures within subjects ANOVA showing serum metabolites commonly altered by exercise in controls and people with type 1 diabetes (T1D). NEFA = Non-esterified Fatty Acid. AC = Acylcarnitine, 5HIAA = 5-hydroxyindoleacetic acid. Adj. *P*-values represent Bonferroni-correction for multiple hypothesis testing. Control n = 30, T1D = 30.

Metabolite	Group (Con, T1D) X Exercise Interaction (F.val)	Group (Con, T1D) X Exercise Interaction (raw.*p*-value)	Group (Con, T1D) X Exercise Interaction (adj.*p*-value)
AC C14:1	12.252	1.60×10^{-5}	0.001345
AC C12	11.055	4.29×10^{-5}	0.0018
AC C12:1	9.7681	0.000126	0.001932
NEFA C20:2	9.6614	0.000138	0.001932
NEFA C16:2	9.8725	0.000115	0.001932
NEFA C18:0	9.9957	0.000104	0.001932
NEFA C20:1	8.8799	0.000269	0.003227
NEFA C17:0	8.5783	0.000349	0.003661
NEFA C22:4	7.1592	0.001203	0.009108
NEFA C22:1	7.0706	0.001301	0.009108
NEFA C22:5	6.2393	0.002727	0.017623
AC C16:1	5.5114	0.005259	0.029448

ESM Table 10. Two-way repeated measures within subjects ANOVA showing serum metabolites exhibiting a differential response to exercise between controls and people with Type 1 Diabetes. NEFA = Non-esterified Fatty Acid. AC = Acylcarnitine. Adj. *p*-values represent Bonferroni-correction for multiple hypothesis testing. Control n = 30, T1D = 30.

Metabolite	F-value	*p*-value	FDR	Significant Group Differences (Tukey's Post-hoc test)
NEFA C22:0	13.661	$8.44{\times}10^{-7}$	$7.34{\times}10^{-5}$	L-C; U-C
lactic acid	8.4156	0.000104	0.004539	L-C; U-C
malic acid	6.8356	0.000526	0.013464	H-C
NEFA C20:0	6.5056	0.000745	0.013464	L-C; U-C
succinate	6.316	0.000912	0.013464	H-C; U-C
BAIBA	6.2988	0.000929	0.013464	L-C; U-C

ESM Table 11. ANOVA of metabolite species differentiating non-diabetes controls and people with Type 1 Diabetes and undetected, low and high residual plasma C-peptide at baseline. NEFA = Non-esterified Fatty Acid. FDR = False Discovery Rate. *p* – value generated by One-Way ANOVA. Significant between group differences calculated using a Tukey's Post-hoc test. Group C = non diabetes control participants (n = 30), group H = high (≥200 pmol/L) residual C-peptide (n = 10), group L = low (3-200 pmol/L) residual C-peptide (n = 9), group U = undetected (<3 pmol/L) C-peptide (n = 11). NEFA = Non-esterified fatty acid, BAIBA = beta-aminoisobutyric acid.

Metabolite	F-value	*p*-value	Significant Group Differences (Tukey's Post-hoc test)
pyruvate	5.7188	0.008	H-U; L-U
AC C14:1	3.5215	0.043	H-U
AC C14	3.4255	0.047	H-U
AC 16:1	3.4049	0.048	H-U
AC 16	3.3853	0.049	H-U
malic acid	3.1459	0.05	H-L: H-U

ESM Table 12. ANOVA of metabolite species differentiating people with Type 1 Diabetes and undetected, low and high residual plasma C-peptide at baseline. *p* – value generated by One-Way ANOVA. Significant between group differences calculated using a Tukey's Post-hoc test. Group H = high (≥200 pmol/L) residual C-peptide (n = 10), group L = low (3-200 pmol/L) residual C-peptide (n = 9), group U = undetected (<3 pmol/L) C-peptide (n = 11). AC = acylcarnitine.

Metabolite	Leverage	SPE
AC C18:1	0.065999	3.0719
NEFA C20:0	0.064358	4.4464
BAIBA	0.061729	8.143
AC C16:1	0.050088	5.9412

ESM Table 13. ANOVA Simultaneous Component Analysis (ASCA) of the interaction between exercise and C-peptide status. Metabolites exhibiting a differential effect to exercise in people with Type 1 Diabetes dependent on C-peptide status are shown. Leverage measures the influence of each observation for a principal component. Score plots will identify observations with high leverages, ie. observations that tend to contribute mostly to separation in the PCA. SPE = squared prediction error. SPE measures the expected squared distance between the predicted value and the true value (i.e. it measures the quality of the predictor). The model was significant as determined by a permutation test statistic of $p < 0.05$. NEFA = Non-esterified Fatty Acid. AC = Acylcarnitine. High (≥200 pmol/L) residual C-peptide (n = 10), low (3-200 pmol/L) residual C-peptide (n = 9), undetected (<3 pmol/L) C-peptide (n = 11).

Metabolite	Correlation	t-stat	*p*-value
NEFA C18:1	-0.44067	-3.7386	0.000425
AC C2	-0.43654	-3.6953	0.000488
NEFA C16:0	-0.43175	-3.6453	0.000572
NEFA C15:0	-0.43052	-3.6326	0.000596
NEFA C17:1	-0.42643	-3.5904	0.00068
NEFA C14:0	-0.42615	-3.5876	0.000686
NEFA C17:0	-0.41064	-3.4298	0.001118
NEFA C18:3	-0.38279	-3.1556	0.002539
NEFA C16:1	-0.37754	-3.1051	0.002941
NEFA C20:3	-0.36822	-3.0162	0.003796
NEFA C20:1	-0.35918	-2.931	0.004827
NEFA C22:5	-0.35166	-2.8609	0.005864
NEFA C22:4	-0.33701	-2.7261	0.008462
NEFA C12:0	-0.33519	-2.7095	0.008845
NEFA C20:5	-0.33021	-2.6642	0.009976
Tryptophan	0.32597	2.6259	0.011033
MOVA	0.32036	2.5756	0.012579
malic acid	-0.31628	-2.5391	0.013819
NEFA C20:2	-0.31316	-2.5113	0.014837
AC C16	-0.29813	-2.3787	0.020688
cis-aconitate	-0.29666	-2.3658	0.021355
tryptamine	0.2962	2.3618	0.021565
AC C3	-0.29058	-2.3128	0.024304
NEFA C18:0	-0.28907	-2.2996	0.025088
NEFA C20:4	-0.27319	-2.1628	0.034691
NEFA C22:6	-0.27241	-2.1561	0.035233
AC C16:1	-0.27236	-2.1557	0.035265
BAIBA	0.25559	2.0134	0.048726

ESM Table 14. Baseline serum metabolites significantly correlated with VO_2Peak in the total study population. Correlation analysis by Pearson *r* showing correlation, t-statistic and *P*-value. NEFA = Non-esterified Fatty Acid; AC = Acylcarnitine; MOVA = methyl-oxo-valeric acid; BAIBA = β-aminoisobutyric acid. N = 60.

Metabolites	Correlation	t-stat	*p*-value
AC C16	-0.61818	-4.1615	0.000272
NEFA C15:0	-0.61534	-4.1307	0.000296
NEFA C17:0	-0.601	-3.979	0.000445
AC C2	-0.59814	-3.9495	0.000481
NEFA C18:1	-0.57431	-3.7123	0.000904
NEFA C16:0	-0.5648	-3.6216	0.001148
Tryptamine	0.54471	3.4369	0.001857
NEFA C20:3	-0.54233	-3.4157	0.001961
NEFA C17:1	-0.54138	-3.4073	0.002005
AC C3	-0.539	-3.3861	0.002117
NEFA C16:1	-0.52926	-3.3008	0.002635
NEFA C22:5	-0.52288	-3.2459	0.003031
NEFA C14:0	-0.5169	-3.1951	0.003447
NEFA C22:4	-0.50225	-3.0734	0.004681
NEFA C20:5	-0.47611	-2.8649	0.007825
NEFA C18:0	-0.46831	-2.8046	0.009053
NEFA C20:4	-0.45746	-2.7222	0.011031
AC C18:1	-0.45598	-2.7111	0.011326
NEFA C22:6	-0.44773	-2.6496	0.0131
AC C18	-0.43954	-2.5894	0.015086
NEFA C20:2	-0.4368	-2.5694	0.015803
NEFA C18:3	-0.41955	-2.4457	0.021
NEFA C20:1	-0.41	-2.3786	0.024435
AC C5:1	-0.40613	-2.3518	0.025951
AC C16:1	-0.39411	-2.2691	0.031161
AC C5	-0.38368	-2.1985	0.036341
AC C8:1	-0.37659	-2.1511	0.040243
Kynurenine	-0.36741	-2.0904	0.045786
Tryptophan	0.35142	1.9863	0.05
BAIBA	0.34826	1.9659	0.05

ESM Table 15. Baseline serum metabolites significantly correlated with *VO_2Peak* in the control population. Correlation analysis by Pearson *r* showing correlation, t-statistic and *p*-value. NEFA = Non-esterified fatty acid; AC = Acylcarnitine; MOVA = methyl-oxo-valeric acid; BAIBA = β-aminoisobutyric acid. N = 30.

ESM Figures

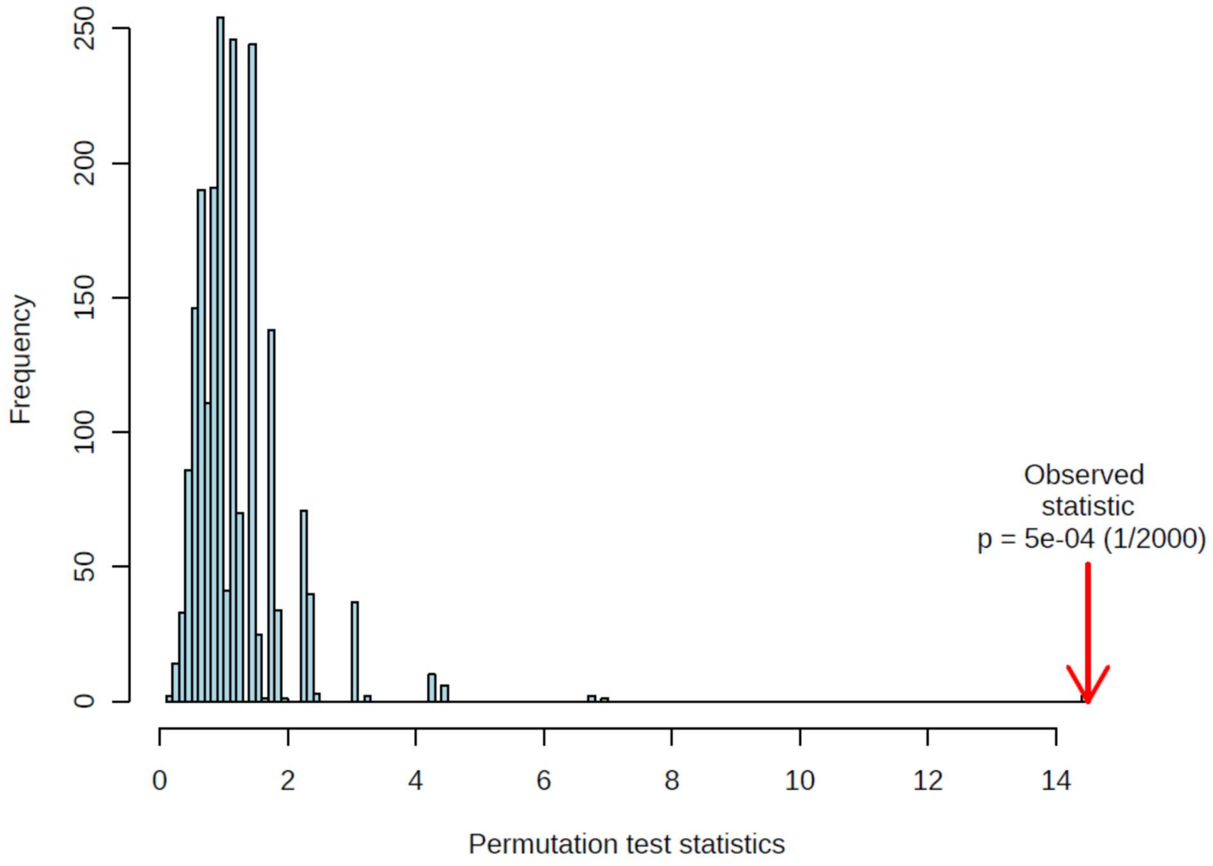

ESM Figure 1. Permutation test statistic for PLS-DA plot of metabolomic data from serum samples at baseline. The distribution formed by randomly permuted data in the permutation test statistic for the PLS-DA model of metabolomic data from serum samples at baseline against the observed statistic for the original model and the number of the 2000 random permutations that outperformed the original data (*p* = 0.0005) (1/2000).

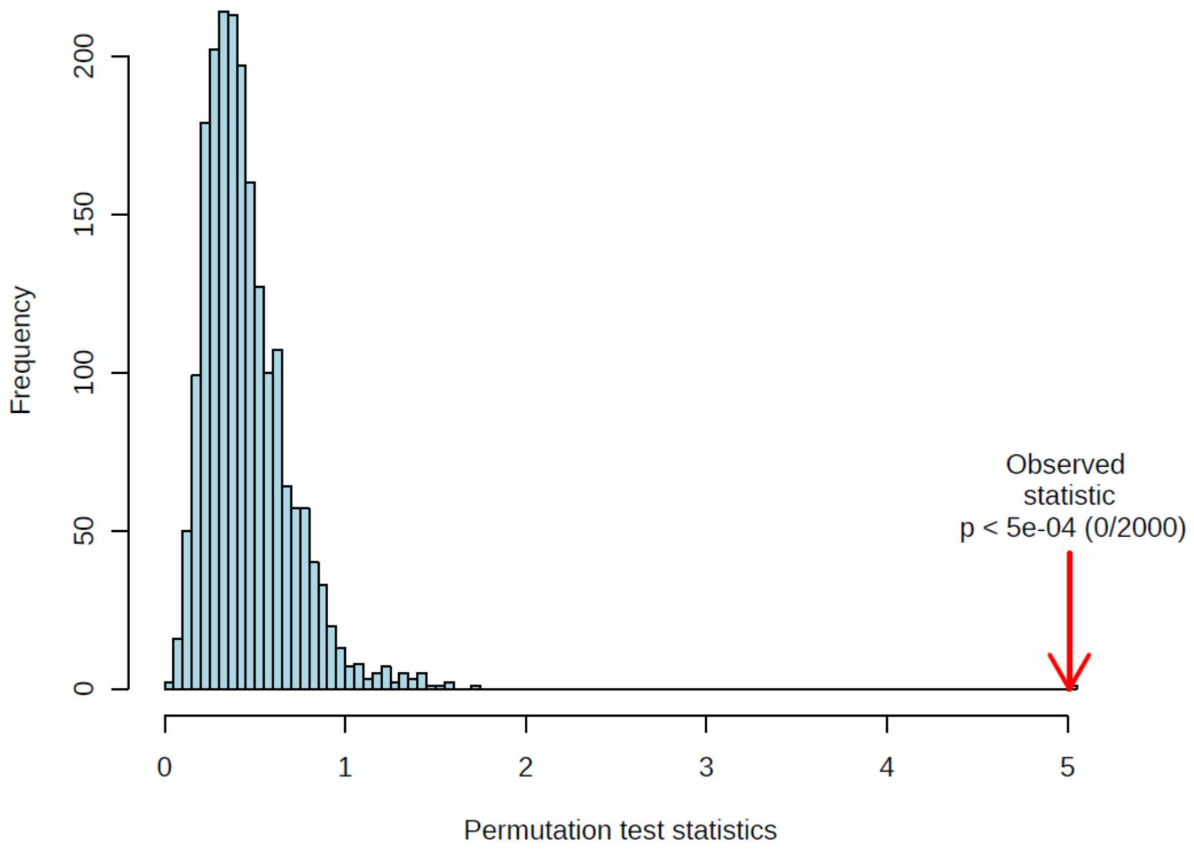

ESM Figure 2. Permutation test statistic for PLS-DA plot of metabolomic data from baseline, exercise and post exercise 1hr recovery of non-diabetes controls. The distribution formed by randomly permuted data in the permutation test statistic for the PLS-DA model of metabolomic data from serum samples from baseline, exercise and post exercise 1hr recovery of non-diabetes controls against the observed statistic for the original model and the number of the 2000 random permutations that outperformed the original data ($p < 0.0005$) (0/2000).

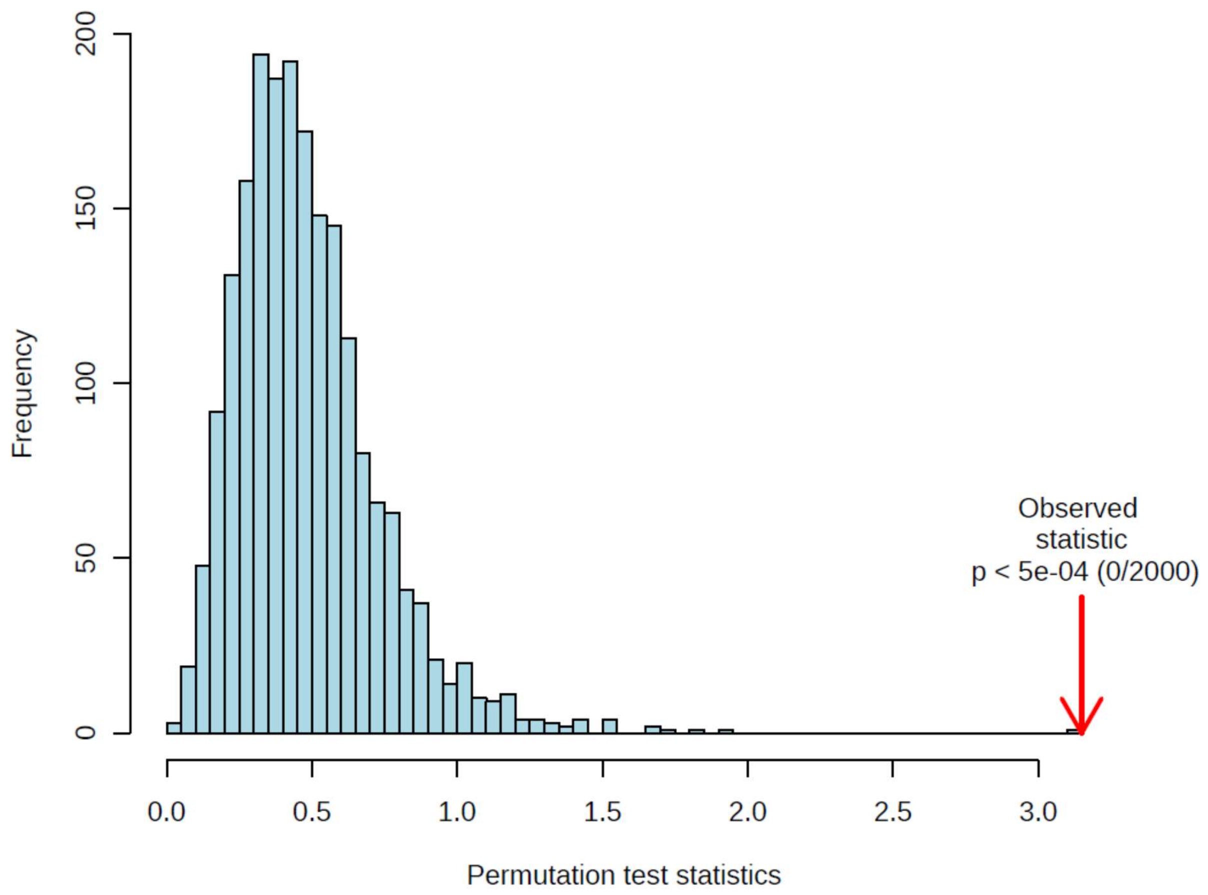

ESM Figure 3. Permutation test statistic for PLS-DA plot of serum metabolomic data from baseline, exercise and post exercise 1hr recovery of people with type 1 diabetes . The distribution formed by randomly permuted data in the permutation test statistic for the PLS-DA model of metabolomic data from serum samples from baseline, exercise and post exercise 1hr recovery of people with type 1 diabetes against the observed statistic for the original model and the number of the 2000 random permutations that outperformed the original data ($p < 0.0005$) (0/2000).

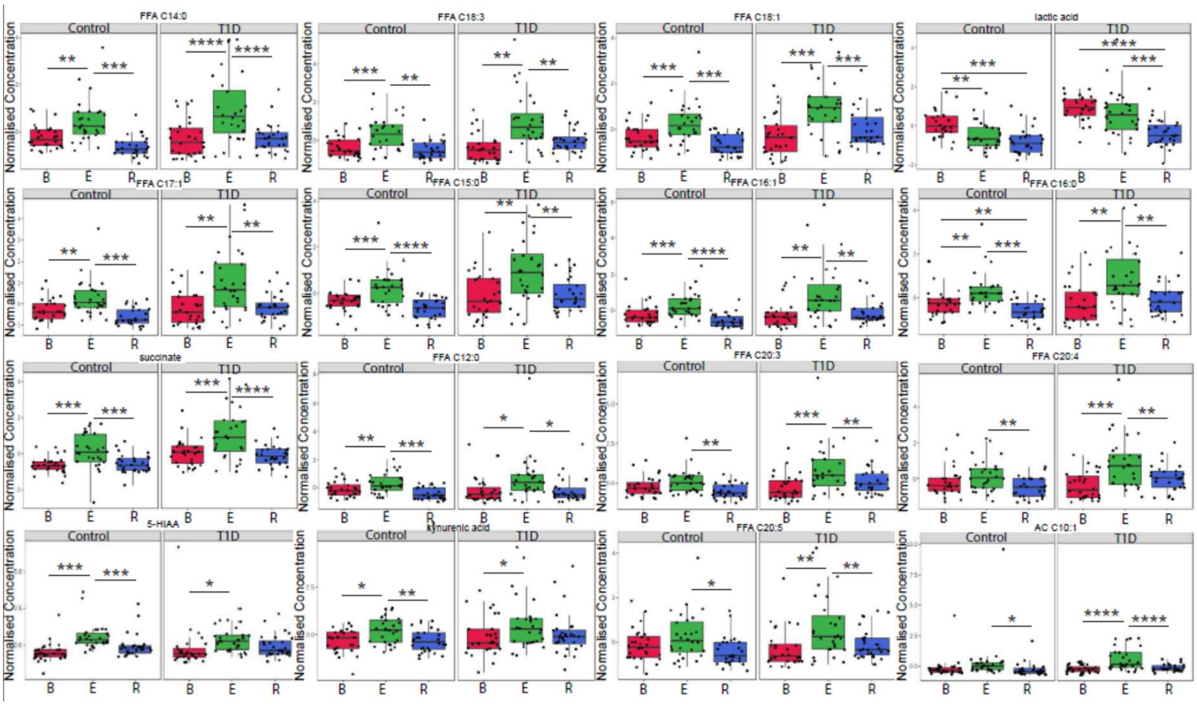

ESM Figure 4. Common metabolic response to aerobic exercise in non-diabetes controls (Control) and people with type 1 diabetes. Metabolite species from baseline (B, red), exercise (E, green) and post exercise 1hr recovery (blue, R) samples with common responses to exercise. Identified with multivariate empirical bayes analysis of variance (MEBA) for time series, which is designed to compare temporal profiles across different biological conditions. Corresponding univariate Two-way within subjects ANOVA data is given in **ESM Table 9**. Data is mean centred. Box and whisker plots show 25th and 75th percentile and the median. Upper whisker = Q3 + 1.5XIQR (Interquantile Range), lower whisker is Q1 - 1.5XIQR. Data is mean centred. FFA = free fatty acid/ non-esterified fatty acid, AC = acylcarnitine, 5HIAA = 5-Hydroxyindoleacetic acid. Control n = 30, T1D = 30. **** $p < 0.0001$, *** $p < 0.001$, ** $p < 0.01$, * $p < 0.05$.

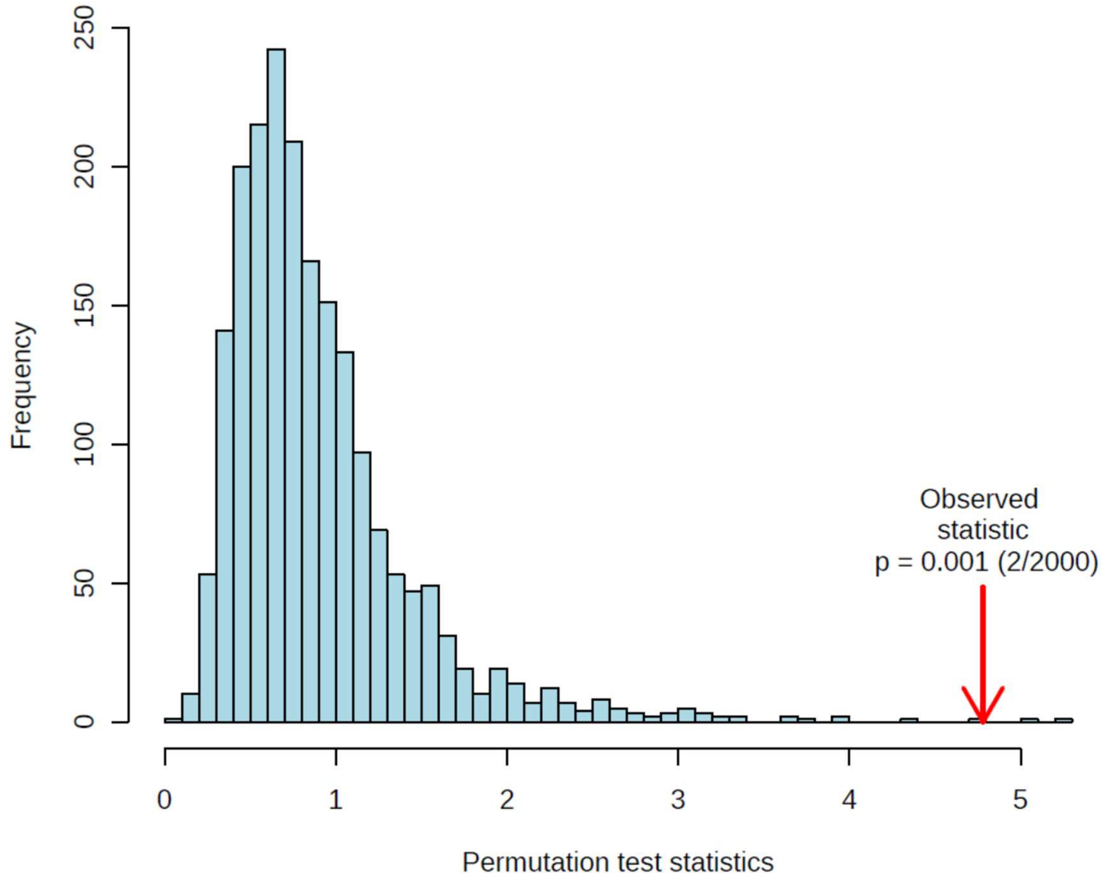

ESM Figure 5. Permutation test statistic for PLS-DA plot of serum metabolomic data from baseline with data grouped according to C-peptide status and compared with control. The distribution formed by randomly permuted data in the permutation test statistic for the PLS-DA model of metabolomic data from serum samples at baseline showing separation of control from people with type 1 diabetes and high plasma C-peptide (≥200 pmol/L), low plasma C-peptide ((3-200 pmol/L) and undetectable plasma C-peptide (<3 pmol/L). (*p* = 0.001) (2/2000).

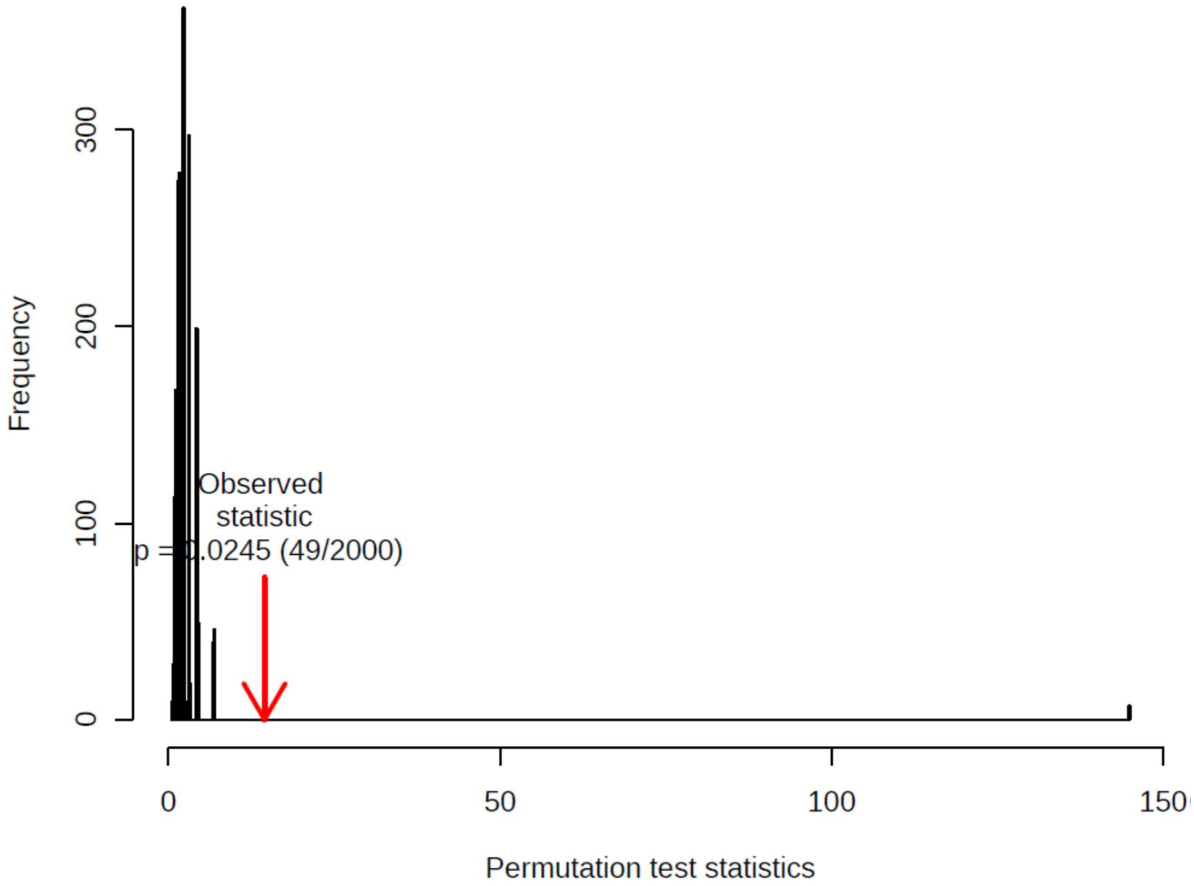

ESM Figure 6. Permutation test statistic for PLS-DA plot of serum metabolomic data from baseline of C-peptide high, low and undetected groups. The distribution formed by randomly permuted data in the permutation test statistic for the PLS-DA model of metabolomic data from serum samples at baseline showing separation of high plasma C-peptide (≥200 pmol/L), low plasma C-peptide ((3-200 pmol/L) and undetectable plasma C-peptide (<3 pmol/L) groups. (*p* = 0.0245) (49/2000).

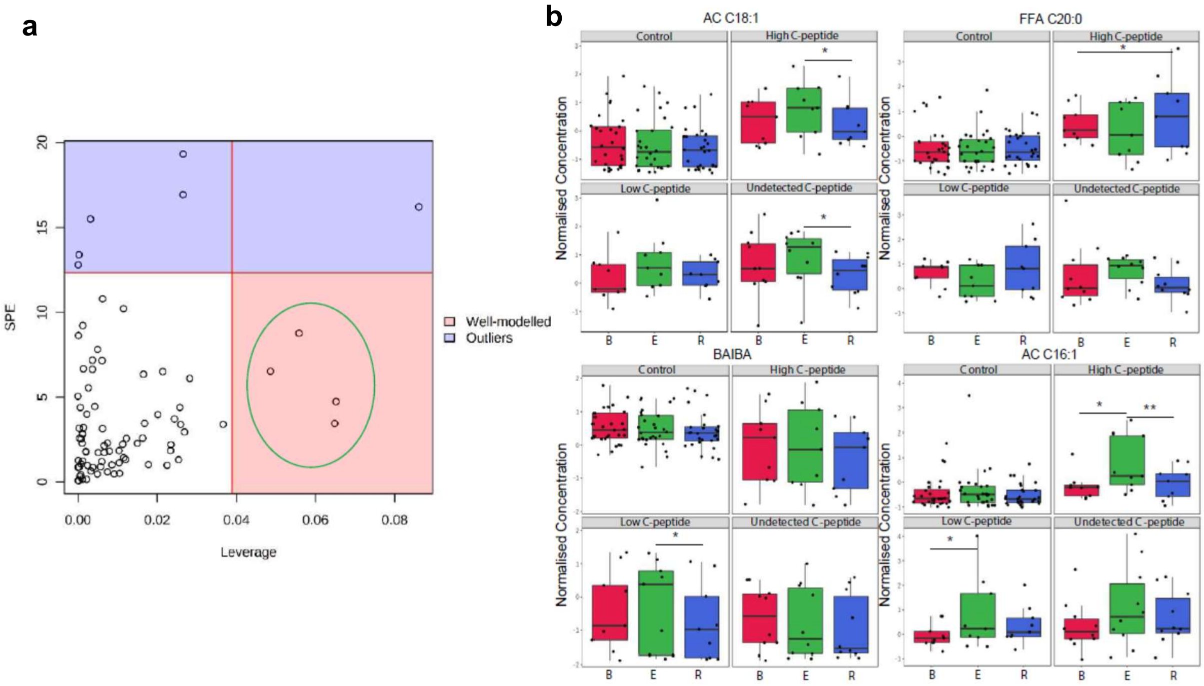

ESM Figure 7. C-peptide status effects the metabolic response to exercise in type 1 diabetes a) An ANOVA Simultaneous Component Analysis (ASCA) plot of the serum metabolomic data for baseline, exercise and 1 hr post exercise recovery in the non-diabetes control and type 1 diabetes volunteers. ASCA identifies major patterns with regard to the c-peptide status and exercise response and their interaction. Metabolites highlighted by the green circle distinguish the C-peptide status groups. Leverage measures the influence of each observation for a principal component. Score plots will identify observations with high leverages, ie. observations that tend to contribute mostly to separation in the PCA. SPE = squared prediction error. SPE measures the expected squared distance between the predicted value and the true value (ie. it measures the quality of the predictor). The model was significant as determined by a permutation test statistic of $p < 0.05$. **b**) Box and whisker plots of the concentration of the 4 metabolites identified by the ASCA model to exhibit a differential effect to exercise in people with T1D dependent on C-peptide status (Control, type 1 diabetes and high plasma C-peptide (≥200 pmol/L), type 1 diabetes and low plasma C-peptide ((3-200 pmol/L) and type 1 diabetes and undetectable plasma C-peptide (<3 pmol/L)). For corresponding Leverage and SPE see **ESM Table 11**. Box and whisker plots show 25th and 75th percentile and the median. Upper whisker = Q3 + 1.5XIQR (Interquantile Range), lower

whisker is Q1 - 1.5XIQR. Data are mean centred. B = baseline serum (red), E = aerobic exercise serum (green), R = 1 hour post exercise recovery serum (blue). FFA = free fatty acid / non-esterified fatty acid, AC = acylcarnitine, BAIBA = beta-aminoisobutyric acid. Non-diabetes controls (n = 30), high (≥200 pmol/L) residual C-peptide (n = 10), low (3-200 pmol/L) residual C-peptide (n = 9), undetected (<3 pmol/L) C-peptide (n = 11). ** $p < 0.01$, * $p < 0.05$.

ESM References

[1] Greenbaum CJ, Mandrup-Poulsen T, McGee PF, et al. (2008) Mixed-meal tolerance test versus glucagon stimulation test for the assessment of beta-cell function in therapeutic trials in type 1 diabetes. Diabetes Care 31: 1966-1971

[2] Palmer JP, Fleming GA, Greenbaum CJ, et al. (2004) C-peptide is the appropriate outcome measure for type 1 diabetes clinical trials to preserve beta-cell function: report of an ADA workshop, 21-22 October 2001. Diabetes 53: 250-264

[3] Bijlsma S, Bobeldijk I, Verheij ER, et al. (2006) Large-scale human metabolomics studies: a strategy for data (pre-) processing and validation. Anal Chem 78: 567-574
